# A SlMYB78‐regulated bifunctional gene cluster for phenolamide and salicylic acid biosynthesis during tomato domestication, reducing disease resistance

**DOI:** 10.1111/jipb.13899

**Published:** 2025-03-28

**Authors:** Peng Cao, Linghao Xia, Xianggui Li, Meng Deng, Zhonghui Zhang, Xiangyu Lin, Zeyong Wu, Yingchen Hao, Penghui Liu, Chao Wang, Chun Li, Jie Yang, Jun Lai, Jun Yang, Shouchuang Wang

**Affiliations:** ^1^ National Key Laboratory for Tropical Crop Breeding, School of Breeding and Multiplication (Sanya Institute of Breeding and Multiplication) Hainan University Sanya 572025 China; ^2^ National Key Laboratory for Tropical Crop Breeding, College of Tropical Agriculture and Forestry Hainan University Sanya 572025 China; ^3^ Yazhouwan National Laboratory Sanya 572025 China

**Keywords:** bifunctional, disease resistance, domestication, gene cluster, phenolamide, salicylic acid, SlMYB78

## Abstract

Plants have evolved a sophisticated chemical defense network to counteract pathogens, with phenolamides and salicylic acid (SA) playing pivotal roles in the immune response. However, the synergistic regulatory mechanisms of their biosynthesis remain to be explored. Here, we identified a biosynthetic gene cluster on chromosome 2 (BGC2) associated with the biosynthesis of phenolamide and SA, wherein the key component *SlEPS1* exhibits dual catalytic functions for the synthesis of phenolamides and SA. Overexpression of the key component *SlEPS1* of BGC2 in tomato enhanced resistance to the bacterial pathogen *Pst DC3000*, whereas knockout plants were more susceptible. Exogenous applications of SA and phenolamides revealed that these two compounds act synergistically to enhance plant resistance. Notably, during tomato domestication, a disease‐resistant allele of *SlEPS1*, *SlEPS1* ^HapB^, was subject to negative selection, leading to a reduction in phenolamide and SA levels and compromised disease resistance in modern varieties. Moreover, the *SlMYB78* directly regulates the BGC2 gene cluster to enhance phenolamide and SA biosynthesis, modulating resistance to *Pst DC3000*. Our study employed multi‐omics approaches to describe the synergistic regulation of phenolamide and SA biosynthesis, offering new insights into the complexity of plant immune‐related metabolism.

## Graphical abstract

The tomato transcription factor SlMYB78 controls a key metabolic gene cluster including *ENHANCED PSEUDOMONAS SUSCEPTIBILTY 1*, encoding a key enzyme that produces disease‐fighting compounds (phenolamides) and immune signals (salicylic acid). During domestication, tomatoes lost a superior variant (*SlEPS1^HapB^*), weakening disease resistance; restoring this variant could reduce pesticide use in agriculture.

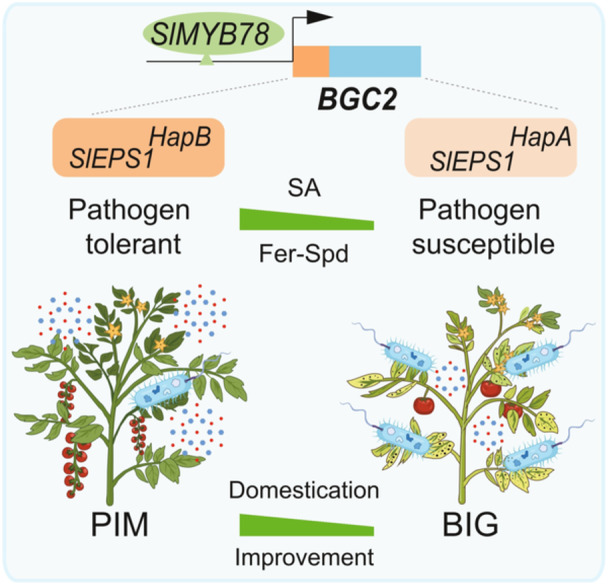

## INTRODUCTION

Tomato (*Solanum lycopersicum*), a widely consumed vegetable crop, is a key model organism in scientific research due to its agricultural significance (Sonawane et al., 2023). However, modern tomato cultivars, particularly those with a focus on high yields, have become more susceptible to pathogens like *Pseudomonas syringae*, leading to reduced quality and yields (Lin et al., 2014; Panno et al., 2021; Lonjon et al., 2024). In contrast, the wild tomato, *Solanum pimpinellifolium* (PIM), an ancestor of both *S. lycopersicum* (BIG) and *Solanum lycopersicum* var. *cerasiforme* (CER), shows significant genetic diversity and has evolved complex chemical defense mechanisms involving metabolites such as phenolamides and salicylic acid (SA) (Zhu et al., 2018; Szymański et al., 2020). These metabolites play pivotal roles in the plant immune response against pathogens (Bassard et al., 2010; Shen et al., 2023).

Phenolamides, which are conjugates of hydroxycinnamic acid derivatives and polyamines (PAs) biosynthesized by hydroxycinnamoyl transferases (HCTs), have well‐documented benefits in plant defense against biotic stresses (D'Auria, 2006; Bassard et al., 2010; Zeiss et al., 2020). For instance, feruloyl putrescine (Fer‐Put) levels rise significantly in rice during brown planthopper infestations and in tobacco plants infected with tobacco mosaic virus (TMV), demonstrating their antimicrobial properties as phytoalexins (Martin‐Tanguy, 1985; Tanabe et al., 2016). Caffeoyl putrescine (Caf‐Put), *p*‐coumaroyl agmatine (Cou‐Agm), and *p*‐coumaroyl putrescine (Cou‐Put) are particularly effective at inhibiting *Phytophthora infestans* growth in potato (Dobritzsch et al., 2016). Moreover, tyramine‐derived phenolamides have been demonstrated to induce strong *Xanthomonas oryzae* resistance in rice (Shen et al., 2021). Despite these findings, the role of phenolamides in bacterial disease tolerance in tomato remains underexplored.

Salicylic acid is a key phytohormone in plant immunity, essential for resistance against biotrophic pathogens like *P. syringae* (Zhou and Zhang, 2020). Mutations in SA biosynthesis genes, such as *EPS1*, *ICS1*, and *PBS3*, impair SA synthesis and increase susceptibility to *P. syringae* (Lee et al., 2007; Nobuta et al., 2007; Zheng et al., 2009). In *Arabidopsis thaliana*, the isochorismate pathway accounts for about 90% of SA biosynthesis, while the benzoate pathway contributes the remaining 10% and operates independently of the phenylalanine pathway (Rekhter et al., 2019; Torrens‐Spence et al., 2019; Wu et al., 2023). In plant pathogen resistance, SA not only activates local resistance responses but also initiates systemic resistance (Jung et al., 2009). For example, after SA induction, NPR1 accumulates in the nucleus and interacts with transcription factors such as TGA and WRKY, regulating the expression of defense genes (e.g., PR1 and PR2) and thereby enhancing systemic resistance (Kesarwani et al., 2007; Zhou and Zhang, 2020). In addition, SA plays a significant role in the synthesis and metabolism of PAs. Differences in PA accumulation, such as those among *eds1‐2*, *ics1‐1*, *mpk6‐2*, and *npr1‐1* mutants, lead to variation in *P. syringae* resistance (Liu et al., 2020; Rossi et al., 2021; Zhang et al., 2023). Pathogen infection can also activate PA synthesis, leading to local SA accumulation and triggering a systemic defense response (Liu et al., 2020). Overexpression of PA metabolism genes, such as *GhPAO*, increases both PA and SA levels, enhancing resistance to fungal pathogens (Mo et al., 2015). Although the interaction between PA and SA is well‐known, the synergistic regulation of phenolamide and SA biosynthesis remains poorly understood.

In this study, we identified a biosynthetic gene cluster on chromosome 2 (BGC2) involved in the biosynthesis of both phenolamides and SA, which contributes to tomato disease resistance. The key gene *SlEPS1* in this cluster catalyzes the production of phenolamides and SA. During tomato domestication and improvement, *SlEPS1* ^HapB^ underwent negative selection, affecting phenolamide and SA levels and disease resistance in modern tomato varieties. We further analyzed regulatory and evolutionary aspects of this gene cluster, providing a theoretical foundation for understanding the synergistic regulation of metabolite diversity and environmental adaptation in tomato.

## RESULTS

### Metabolome genome‐wide association studies identification of a gene cluster associated with phenolamide and SA biosynthesis

**Figure 1:**
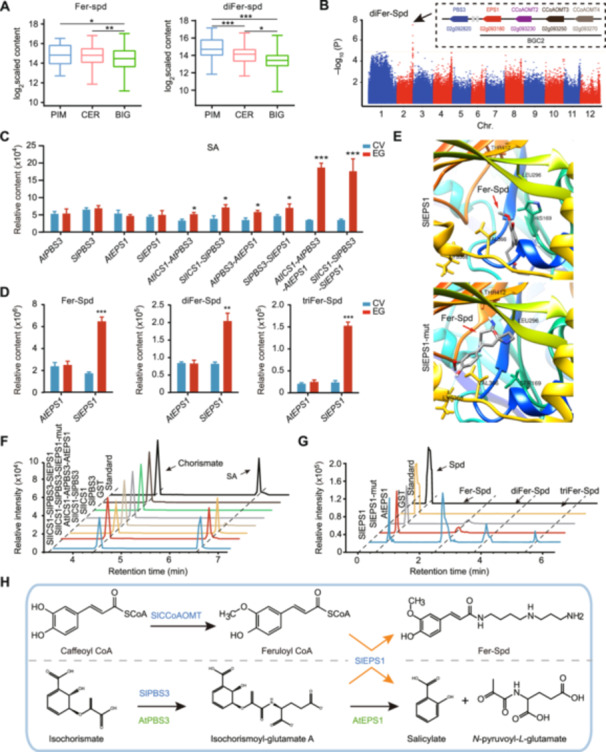
**Identification of the bifunctional enzyme activity of** ***SlEPS1*** **(A)** Boxplots depict the feruloyl – spermidine (Fer‐Spd) and diFer‐Spd content in *Solanum pimpinellifolium* (PIM), *S. lycopersicum* var. *cerasiforme* (CER), and *S. lycopersicum* (BIG) tomato subgroups. The central line within each box represents the median. Significant differences were identified using Student's *t*‐tests. **(B)** A Manhattan plot displaying genome‐wide association study (GWAS) results for the diFer‐Spd content (*n* = 401). Negative log_10−_ transformed *P*‐values from a genome‐wide scan are plotted across the 12 tomato chromosomes. The horizontal dashed line indicates the genome‐wide significance threshold. The genetic map of chromosome 2 illustrates the distribution of candidate metabolic genes within biosynthetic gene cluster on chromosome 2 (BGC2). **(C)** Accumulation of salicylic acid (SA) across various coinfiltration experiments in *Nicotiana benthamiana*. CV, control group. EG, experimental treatment group. **(D)** Accumulation of Fer‐Spd, diFer‐Spd, and triFer‐Spd in *N. benthamiana* leaves transiently overexpressing *AtEPS1* and *SlEPS1*, respectively, with control vector CK. **(E)** The Fer‐Spd‐bound SlEPS1 and SlEPS1‐mut structures. **(F)** High‐performance liquid chromatography (HPLC) chromatograms of SA synthesis catalyzed by recombinant SlICS1, SlPBS3, SlEPS1, SlEPS1‐Mut, AtICS1, AtPBS3, and AtEPS1. Purified glutathione S‐transferase (GST) protein was used as a control. **(G)** HPLC for recombinant SlEPS1, SlEPS1‐Mut, and AtEPS1 with Spd and Fer‐CoA. Purified GST protein was used as the control. **(H)** Metabolic pathways for Fer‐Spd and SA biosynthesis in tomato and Arabidopsis. All experiments were repeated twice with similar results. Data are shown as means ± *SD*, *n* = 3. *P < 0.05, **P < 0.01, and ***P < 0.001 (Student's *t*‐tests).

To elucidate the genetic basis of natural variation in phenolamide levels in tomato, we quantified phenolamide levels in the leaves of 401 core tomato accessions, including 258 BIG, 115 CER, and 28 PIM accessions (Table S1) (Zhu et al., 2018). The majority of phenolamides exhibited a progressive decline in abundance during tomato domestication and improvement (Figure S1). Notably, the concentrations of Fer‐Spd (spermidine) and diFer‐Spd in both CER and PIM cultivars were markedly higher compared to those in BIG varieties (Figure 1A). Next, we performed a genome‐wide association study (GWAS) using 2,318,614 single nucleotide polymorphisms (SNPs) with minor allele frequencies above 0.05, identifying a significant SNP on chromosome 2 associated with diFer‐Spd levels (SF0254126808, *P* = 3.09 × 10^−8^) (Figure 1B). Four genes were found in proximity to this SNP and were correlated with phenolamide levels (Figure 1B). These include an acyltransferase gene, *Solyc02g093180* (*SlEPS1*), which is highly homologous to *Arabidopsis thaliana* acyltransferase EPS1, a key enzyme involved in SA biosynthesis (Figure S2A), and three methyltransferases, *Solyc02g093230* (*SlCoAOMT2*), *Solyc02g093250* (*SlCoAOMT3*), and *Solyc02g093270* (*SlCoAOMT4*), associated with genes involved in feruloyl‐CoA synthesis (Figure S2B). The close proximity of these genes suggests the presence of BGC2, regulating the abundance of SA and Spd‐derived phenolamides in tomato. In addition, a gene related to the SA biosynthetic pathway, *SlPBS3* (*Solyc02g092820*), was located 324 kb upstream of *SlEPS1* (Figure 1B).

Given the high homology between SlEPS1, a core component of BGC2, and AtEPS1, we hypothesized that *SlEPS1* might be involved in SA biosynthesis. We established a co‐expression system (*ICS1*‐*PBS3*‐*EPS1*) for SA biosynthesis in *Nicotiana benthamiana* and found that the expression of *AtEPS1*, *AtPBS3*, *SlEPS1*, or *SlPBS3* alone did not lead to the synthesis of SA (Figure 1C). However, when co‐expressed, combinations such as *AtICS1‐AtPBS3*, *AtPBS3‐AtEPS1*, *SlICS1‐SlPBS3*, or *SlPBS3‐SlEPS1* resulted in a slight increase in SA levels was observed (Figure 1C). Importantly, co‐expression of *SlICS1‐SlPBS3‐SlEPS1* resulted in the significant accumulation of SA, consistent with the results observed for *AtICS1‐AtPBS3‐AtEPS1* (Figure 1C). These experiments in *N. benthamiana* highlight the critical role of *SlEPS1* in SA biosynthesis. Subsequent transient expression experiments in *N. benthamiana* further confirm that *SlEPS1* can catalyze the reaction between feruloyl‐CoA (Fer‐CoA) and spermidine, leading to the accumulation of Fer‐Spd, diFer‐Spd, and triFer‐Spd (Figure 1D). In contrast, *AtEPS1* did not exhibit this activity, supporting the dual role of *SlEPS1* in the biosynthesis of both phenolamides and SA (Figure 1D).

To investigate the reasons for the differences between these two proteins, we conducted interspecies phylogenetic tree analysis and found that EPS1 is conserved in species after land colonization evolution (Figure S3A). Furthermore, multiple sequence alignment analysis revealed that the key amino acid residue in the catalytic active center (HXXXD motif of BAHD acyltransferases) of AtEPS1 is Ser160, while in SlEPS1 it is His169 (Figure S3B). We hypothesize that the histidine (His169) at position 169 in SlEPS1 is crucial for the synthesis of phenolamides and is the key amino acid residue that confers dual functionality to SlEPS1. The structures of both SlEPS1^His169^ and SlEPS1‐mut^Ser169^ were predicted using AlphaFold2 and we performed molecular docking with Fer‐Spd. The results showed that the His169 residue of SlEPS1 can catalyze the synthesis of Fer‐Spd through hydrogen bonding, whereas this phenomenon is absent in SlEPS1‐mut (Figure 1E).

Next, we expressed and purified SlEPS1, SlEPS1‐mut, SlPBS3, SlICS1, AtICS1, AtPBS3, and AtEPS1 in *Escherichia coli* BL21, and confirmed through enzyme activity assays that both the SlEPS1‐SlPBS3‐SlICS1 and AtICS1‐AtPBS3‐AtEPS1 complexes consistently catalyze the formation of SA from chorismic acid (Figure 1F). Furthermore, we observed no significant difference in the synthesis of SA between SlEPS1‐SlPBS3‐SlICS1 and SlEPS1‐SlPBS3‐SlEPS1‐mut (Figure 1F), indicating that the mutation site is not a key active residue for SA biosynthesis. Enzyme activity assays further demonstrated that SlEPS1 can catalyze the formation of Fer‐Spd, diFer‐Spd, and triFer‐Spd from Fer‐CoA and spermidine, highlighting its significant catalytic activity in the biosynthesis of spermidine‐derived phenolamides (Figure 1G). In contrast, co‐incubation of AtEPS1 with Fer‐CoA and spermidine produced only low levels of Fer‐Spd (Figure 1G). Moreover, the activity of SlEPS1‐mut was similar to that of AtEPS1, producing only low levels of Fer‐Spd (Figure 1G), confirming that His160 is the key residue for the catalytic activity of the dual‐function enzyme SlEPS1 in phenolamide synthesis (Figure 1H).

To investigate the differences in resistance to *P. syringae* pv. tomato DC3000 (*Pst* DC3000) between *SlEPS1* and *SlEPS1‐mut*, we transiently expressed *SlEPS1* and *SlEPS1‐mut* in *N. benthamiana* and inoculated the plants with *Pst* DC3000. Both *SlEPS1* and *SlEPS1‐mut* conferred significantly higher resistance compared to the control group, with *SlEPS1* exhibiting a more pronounced effect (Figure S4A, B). This suggests that the dual enzymatic activity of *SlEPS1* is associated with enhanced disease resistance.

### *SlEPS1*‐mediated accumulation of Fer‐Spd and SA synergistically enhances tomato defenses against bacterial pathogens

**Figure 2:**
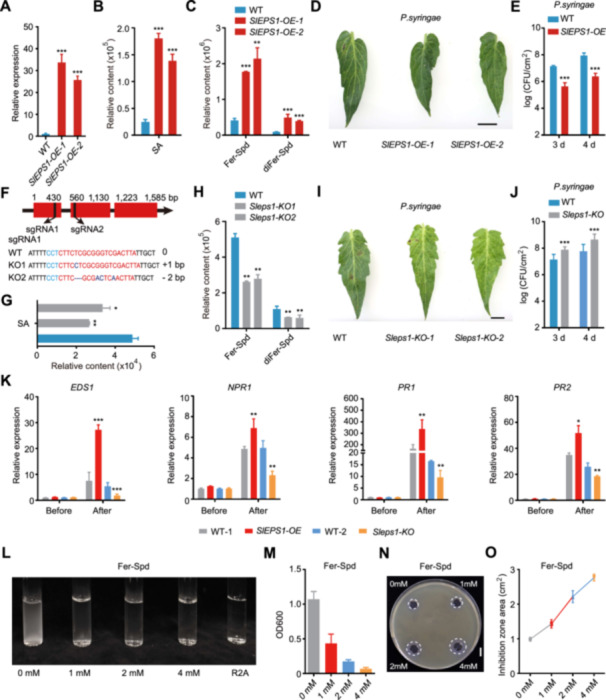
The role of SlEPS1 in tomato disease resistance **(A)** Expression analysis in *SlEPS1‐OE* lines with WT tomato plants as the control. OE, overexpression lines; WT, wild‐type. **(B**, **C)** Bar plots of the salicylic acid (SA) **(B)**, and feruloyl – spermidine (Fer‐Spd) and diFer‐Spd **(C)** content in *SlEPS1‐OE* lines with WT tomato plants as the control. **(D**, **E)** WT plants were more susceptible to *Pst DC3000* infection than *SlEPS1‐OE* plants **(D)**. Bacterial growth on inoculated tomato leaves was quantified at the time points depicted **(E)**. Inoculation occurred with *Pseudomonas syringae* bacteria at 10^5^ colony‐forming units (CFU)/mL (*n* = 12). **(F)** Generation of *SlEPS1* mutants via clustered regularly interspaced short palindromic repeats (CRISPR)/CRISPR‐associated protein 9 (Cas9). Sequences are provided for the *SlEPS1* mutants KO1 and KO2. The single‐guide RNA (sgRNA) targets and a protospacer‐adjacent motif (PAM) are indicated in red and in green, respectively. Deletions and insertions are indicated by dashes and in blue, respectively. KO, CRISPR/Cas9 mutant lines. **(G**, **H)** Bar plots of the SA **(G)**, and Fer‐Spd and diFer‐Spd **(H)** content in *Sleps1‐KO* lines with WT tomato plants as the control. **(I**, **J)** *Sleps1‐KO* lines were more susceptible to *Pst DC3000* infection than WT plants **(I)**. Bacterial growth on inoculated tomato leaves was quantified at the depicted time points **(J)**. Inoculation was conducted using *P. syringae* bacteria at 10^5^ CFU/mL (*n* = 12). **(K)** Expression analysis for *EDS1, NPR1, PR1*, and *PR2* in *SlEPS1‐OE* and *Sleps1‐KO* lines under control conditions and following inoculation with *Pst DC3000*. **(L**, **M)** Representative photographs **(L)** and relative growth **(M)** of *Pst DC3000* cultured with different concentrations of Fer‐Spd. *Pst DC3000* bacteria were cultured in R2A medium supplemented with 0, 1, 2, or 4 mmol/L Fer‐Spd. **(N**, **O)** Representative photographs **(N)** and relative growth **(O)** of *Pst DC3000* illustrating the inhibitory effect of different concentrations of Fer‐Spd on *Pst DC3000* growth when cultured in King B agar. Four Fer‐Spd concentrations were tested: 0, 1, 2, and 4 mmol/L Fer‐Spd. Data are shown as means ± *SD*, *n* = 3. *P < 0.05, **P < 0.01, and ***P < 0.001 (Student's *t*‐tests).

To investigate how *SlEPS1* functions in plant disease resistance, we generated *SlEPS1* overexpression transgenic tomato lines, named *SlEPS1‐OE* (Figure 2A). Metabolite analysis revealed that the overexpression lines accumulated higher levels of Fer‐Spd, diFer‐Spd, and SA compared to wild‐type (WT) plants (Figure 2B, C). Next, we performed bacterial inoculation experiments using *Pst DC3000* on these transgenic tomato plants. Four days post‐inoculation, the *SlEPS1‐OE* lines exhibited only mild disease symptoms, in contrast to the severe lesions observed in WT plants (Figure 2D). Quantification of bacterial growth showed significantly lower levels of *Pst DC3000* in *SlEPS1‐OE* lines compared to WT plants, indicating that overexpression of *SlEPS1* significantly enhances tomato leaf resistance to *Pst DC3000* (Figure 2E). In addition, clustered regularly interspaced short palindromic repeats (CRISPR) was used to generate mutant lines of *SlEPS1*, including *Sleps1‐KO1* (insertion of one base A) and *Sleps1‐KO2* (deletion of two bases T and C) (Figure 2F). Both mutations resulted in premature termination of the protein at amino acid residue 85. The levels of Fer‐Spd, diFer‐Spd, and SA were lower in *Sleps1‐KO* lines as compared to the WT plants (Figure 2G, H). Further inoculation experiments revealed that the *Sleps1‐KO* lines were highly susceptible to *Pst DC3000* (Figure 2I). This finding was corroborated by quantitative data showing increased bacterial growth in the *Sleps1‐KO* lines compared to in WT plants (Figure 2J).

Using quantitative real‐time polymerase chain reaction (qRT‐PCR), we analyzed the expression of genes involved in downstream immune responses in *SlEPS1* transgenic tomato plants. The transcription of defense signaling genes, including *SlEDS1*, *SlNPR1*, *SlPR1*, and *SlPR2*, was significantly upregulated in *SlEPS1‐OE* lines compared to WT plants (Figure 2K). In contrast, transcription of these genes was downregulated in the *Sleps1‐KO* lines (Figure 2K). These data suggest that overexpression of *SlEPS1* enhances tomato resistance to *Pst DC3000*, and that the accumulation of Fer‐Spd, diFer‐Spd, and SA plays a critical role in defending against *Pst DC3000* infection.

To determine the interplay between phenolamides and SA in influencing disease resistance in tomato plants, we conducted a series of experiments. Wild‐type plants were treated with different concentrations of Fer‐Spd (0.2 mmol/L, 0.5 mmol/L, and 1 mmol/L), and the SA content in leaves was measured at various time points post‐treatment. The results showed that SA accumulation initially increased and then declined, peaking at 6 h post‐treatment, with the slowest decrease observed in leaves treated with 1 mmol/L Fer‐Spd (Figure S5A). Conversely, WT plants were treated with varying concentrations of SA (0.1 mmol/L, 0.2 mmol/L, and 0.3 mmol/L) to examine its effect on phenolamide synthesis. Phenolamide levels also followed an initial rise and subsequent fall, reaching a maximum at 12 h post‐treatment, with more stable accumulation in leaves treated with 0.2 mmol/L SA (Figure S5B). We further analyzed the expression of key genes in the SA biosynthesis pathway (*ICS1*, *EDS5*, and *PBS3*) using qRT‐PCR after 1 mmol/L Fer‐Spd treatment, observing significant induction at 6 h (Figure S5C). Similarly, key genes in the phenolamide biosynthesis pathway (*PAL1*, *4CL1*, and *EPS1*) were significantly induced after 0.2 mmol/L SA treatment, with their expression patterns correlating with the trends in phenolamide accumulation (Figure S5D). Pre‐treatment of WT plants with 0.2 mmol/L SA and 1 mmol/L Fer‐Spd for 6 h significantly enhanced resistance to *Pst DC3000* (Figure S5E, F). Furthermore, the relatively low accumulation of SA following the application of 0.2 mmol/L Fer‐Spd allowed for an independent assessment of the antimicrobial efficacy of Fer‐Spd without interference from SA accumulation. Consequently, after treating the plants with 0.2 mmol/L Fer‐Spd followed by inoculation with *Pst DC3000*, we observed that Fer‐Spd significantly enhanced the disease resistance of tomato leaves, although this enhancement was somewhat less pronounced compared to the leaves treated with the same concentration of SA (Figure S5E, F). These findings indicate that phenolamides and SA mutually affect each other's synthesis and work synergistically to enhance disease resistance in tomato.

We hypothesized that Fer‐Spd may inhibit the growth of *Pst DC3000* and alleviate disease symptoms at specific concentrations. To test this hypothesis, we treated *Pst DC3000* with different concentrations of Fer‐Spd in liquid R2A culture medium. The results showed that Fer‐Spd effectively inhibited the growth of *Pst DC3000* in the R2A medium (Figure 2L, M). At a Fer‐Spd concentration of 2 mmol/L, the growth of *Pst DC3000* was completely inhibited (Figure 2L, M). Consistent results were obtained from solid media experiments (Figure 2N, O), demonstrating that Fer‐Spd directly act on bacterial pathogens to inhibit their growth, with a half‐maximal inhibitory concentrations (IC_50_) of 1 mmol/L for Fer‐Spd.

### *SlEPS1* haplotype shifts during tomato domestication reduced resistance to bacterial infections

**Figure 3:**
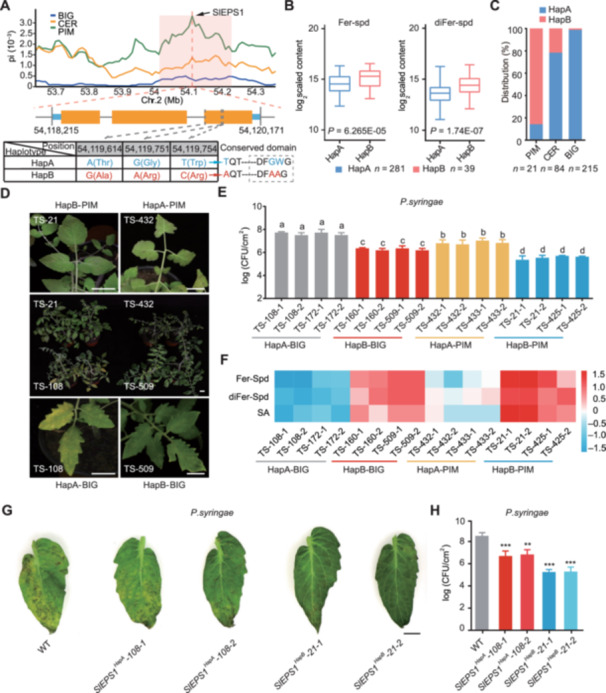
**Stepwise selection of** ***SlEPS1*** **in tomato evolution** **(A)** Nucleotide diversity around the *SlEPS1* locus for three groups of tomato accessions (*Solanum pimpinellifolium* (PIM), *S. lycopersicum* var. *cerasiforme* (CER), and *S. lycopersicum* (BIG)). Three single nucleotide polymorphisms (SNPs) were identified within the coding region of *SlEPS1*. Based on these loci, the three tomato subgroups, BIG, CER, and PIM, were further divided into two combined haplotypes: HapA (*SlEPS1‐A‐G‐T*) and HapB (*SlEPS1‐G‐A‐C*), represented by red and blue at the same site, respectively. **(B)** Boxplots of the Fer‐Spd and diFer‐Spd content in each haplotype group, where *n* indicates the number of accessions belonging to each haplotype. **(C)** Proportion of each *SlEPS1* haplotype across groups of tomato accessions (PIM, CER, and BIG); *n* indicates the number of accessions. **(D)** Evaluation of disease resistance in several tomato varieties and populations carrying different *SlEPS1* haplotypes. Photographs were taken 1 week after inoculation with *Pst DC3000*. Scale bar = 2 cm. **(E)** Growth of *Pst DC3000* in tomato varieties with distinct *SlEPS1* haplotypes across populations as quantified 4 d after inoculation. Inoculation utilized *Pst. DC3000* at 10^5^ colony‐forming units (CFU)/mL (*n* = 12). **(F)** Metabolite analysis for tomato varieties with different *SlEPS1* haplotypes across populations. Metabolite data were Z‐score standardized to range from −1.5 to 1.5 per metabolite. The color scale represents the log^2^ of the relative mass spectral intensity values. **(G)** Assessment of disease resistance in *SlEPS1* ^HapA^ *‐108* and *SlEPS1* ^HapB^ *‐21* transgenic plants. Photographs were taken 1 week after inoculation with *Pst DC3000*. Scale bar = 1 cm. **(H)** Comparisons of bacterial growth among *SlEPS1* ^HapA^ *‐108* and *SlEPS1* ^HapB^ *‐21* transgenic plants at 4 d after inoculation with *Pst DC3000*. All experiments were repeated twice with similar results. Data are shown as the mean ± *SD*, *n* = 3, **P < 0.01 and ***P < 0.001; Student's *t*‐tests.

To understand how tomato domestication and improvement have affected *SlEPS1*, we compared the nucleotide diversity (*π*) of three tomato subgroups, including 215 BIG, 84 CER, and 21 PIM accessions (Zhu et al., 2018). Nucleotide diversity at the *SlEPS1* locus progressively decreased from PIM to CER and then to BIG, suggesting that this gene may have been affected by artificial selection (Figure 3A). Resequencing of 320 accessions revealed three nonsynonymous mutations (G/A; A/G; C/T) in the third exon of the *SlEPS1* coding sequence (CDS), which were significantly associated with the Fer‐Spd and diFer‐Spd content (Table S4) (Zhu et al., 2018). Based on these variants, the tomato population was divided into two haplotype groups (HapA and HapB) (Figure 3A). We found that Fer‐Spd and diFer‐Spd levels in plants carrying *SlEPS1* ^HapA^ were significantly lower than in those carrying *SlEPS1* ^HapB^ (*P* = 6.265 × 10^−5^; *P* = 1.74 × 10^−7^) (Figure 3B). The frequency of *SlEPS1* ^HapA^ gradually increased during tomato domestication and improvement, becoming most common in the BIG subgroup, while the majority of PIM accessions carried *SlEPS1* ^HapB^ (Figure 3C). Therefore, we initially classified the *SlEPS1* ^HapB^ haplotype as disease‐resistant (high phenolamide content) and the *SlEPS1* ^HapA^ haplotype as susceptible (low phenolamide content).

To further investigate the role of *SlEPS1* haplotypes in tomato disease resistance, we selected 10 representative accessions and evaluated their disease resistance. These accessions included two BIG accessions carrying *SlEPS1* ^HapA^ (TS‐108 and TS‐172), three BIG accessions carrying *SlEPS1* ^HapB^ (TS‐74, TS‐160, and TS‐509), two PIM accessions carrying *SlEPS1* ^HapA^ (TS‐432 and TS‐433), and three PIM accessions carrying *SlEPS1* ^HapB^ (TS‐425, TS‐415, and TS‐21). Our research demonstrates that PIM accessions exhibit significantly greater disease resistance compared to BIG accessions. Moreover, accessions carrying *SlEPS1* ^HapB^ were significantly more resistant than those carrying *SlEPS1* ^HapA^ (Figure S6). For example, the TS21 variety, characterized by smaller fruits and smooth leaves, exhibited significantly higher disease resistance compared to the TS108 variety, which has larger fruits and rough leaves (Figure 3D). In addition, in accessions carrying *SlEPS1* ^HapB^, the growth of the pathogen was more effectively inhibited (Figure 3E). Metabolomic analysis revealed the levels of Fer‐Spd, diFer‐Spd, and SA were higher in lines carrying *SlEPS1* ^HapB^ compared to those carrying *SlEPS1* ^HapA^ (Figure 3F). Collectively, these results suggest that natural variation in *SlEPS1* is closely related to differences in phenolamide and SA content, thereby influencing plant resistance.

To validate the function of different *SlEPS1* haplotypes, we expressed and purified SlEPS1^HapB^ (from TS‐21) and SlEPS1^HapA^ (from TS‐108) in *E. coli* BL21, named SlEPS1^HapB^‐21 and SlEPS1^HapA^‐108, respectively. Enzyme activity assays showed that SlEPS1^HapA^ had significantly lower catalytic activity for both SA and Fer‐Spd compared to SlEPS1^HapB^ (Figure S7A, B), suggesting that natural variations in SlEPS1 can simultaneously affect the levels of SA and Fer‐Spd. Next, we transiently expressed *SlEPS1* ^HapB^‐21 and *SlEPS1* ^HapA^‐108 in *N. benthamiana* and in *VF36* tomato varieties, respectively. After inoculating with *Pst DC3000*, the *N. benthamiana* and *VF36* tomato leaves transiently expressing either *SlEPS1* haplotype exhibited enhanced resistance compared to controls (Figure S7C, E). This was particularly true for *SlEPS1* ^HapB^‐21, which showed the lowest bacterial growth after inoculation (Figure S7D, F), indicating that *SlEPS1* ^HapB^ confers strong *Pst DC3000* resistance in *N. benthamiana* and tomato leaves. To further validate this finding, we introduced *SlEPS1* ^HapB^‐21 and *SlEPS1* ^HapA^‐108 into MicroTom tomato varieties. In lines with comparable expression levels, the levels of SA, Fer‐Spd, and diFer‐Spd were significantly higher in the *SlEPS1* ^HapB^‐21 lines than in the *SlEPS1* ^HapA^‐108 lines (Figure S7G–I). Disease resistance evaluation indicates that although *SlEPS1* ^HapA^‐108 plants exhibited greater resistance than WT plants, *SlEPS1* ^HapB^‐21 plants displayed significantly higher resistance than *SlEPS1* ^HapA^‐108 plants (Figure 3G, H), confirming that *SlEPS1* ^HapB^ confers stronger resistance to *Pst DC3000*. In summary, *SlEPS1* ^HapB^ was negatively selected during tomato domestication and improvement, leading to reductions in the phenolamide and SA content and in disease resistance in modern cultivars. We propose that selecting for *SlEPS1* ^HapB^ may help improve tomato disease resistance.

### Evolutionary history of the tomato biosynthetic gene cluster BGC2

**Figure 4:**
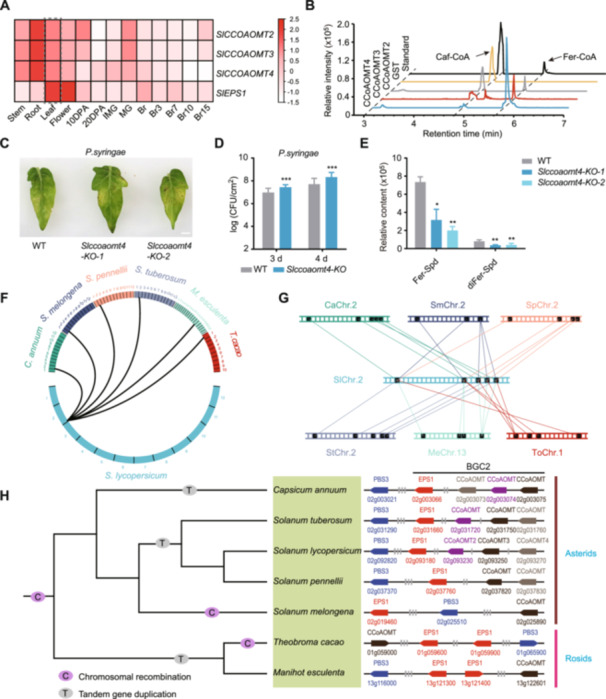
***SlEPS1*** **contributed to the formation and evolution of the biosynthetic gene cluster on chromosome 2 (BGC2) in tomato** **(A)** Expression profiles for BGC2 genes in different tomato tissues. **(B)** High‐performance liquid chromatography (HPLC) chromatograms for recombinant SlCoAOMT2, SlCoAOMT3, and SlCoAOMT4, with Caf‐CoA. Purified glutathione S‐transferase (GST) protein was used as the control. **(C**, **D)** *Slcoaomt4‐KO* lines were more susceptible to *Pst DC3000* infection than wild‐type (WT) plants **(C)**. Bacterial growth of inoculated tomato leaves was quantified at the depicted time points **(D)**. Inoculation was carried out with *Pst. DC3000* bacteria at 10^5^ colony‐forming units (CFU)/mL (*n* = 12). **(E)** Bar plots of the feruloyl – spermidine (Fer‐Spd) and diFer‐Spd content in *Slcoaomt4‐KO* lines with wild‐type (WT) tomato plants as the control. **(F)** Gene syntenic regions identified across tested plant species. Solid lines represent syntenic regions within the BGC2–SlPBS2 module. **(G)** Synteny diagram for the BGC2–SlPBS2 module across tested plant species. **(H)** Schematic diagram of evolutionary events affecting the BGC2–SlPBS2 module. All experiments were repeated twice with similar results. Data are shown as the mean ± *SD*, *n* = 3, *P < 0.05, **P < 0.01 and ***P < 0.001; Student's *t*‐tests.

We analyzed the expression of the three other genes in the BGC2 cluster (*SlCoAOMT2, SlCoAOMT3*, and *SlCoAOMT4*) and found they were highly co‐expressed with *SlEPS1* in tomato leaves (Figure 4A). Furthermore, a co‐expression analysis for core genes in the phenolamide and SA biosynthetic pathways revealed significant co‐expression and strong correlations between the BGC2 genes and those involved in phenolamide biosynthesis (such as the *SlPAL* and *Sl4CL* family genes), as well as those involved in SA biosynthesis (such as *SlICS1* and *SlEDS5*) (Figure S8). We then cloned the CDS of *SlCoAOMT2*, *SlCoAOMT3*, and *SlCoAOMT4*, expressed and purified them. When incubated with Caf‐CoA and S‐adenosylmethionine (SAM), these enzymes catalyzed the formation of Fer‐CoA, confirming their function as Fer‐CoA synthases (Figure 4B).

To assess the response of BGC2 gene expression after infection with *Pst DC3000*, we quantified the transcription of the BGC2 genes at different time points after inoculation. Gene expression initially increased and then decreased, peaking at 8 h after treatment (Figure S9). This suggests that, in addition to *SlEPS1*, *SlCoAOMT2*, *SlCoAOMT3*, and *SlCoAOMT4* may also play roles in responding to *Pst DC3000* infection. To further explore the role of *SlCoAOMT2*, *SlCoAOMT3*, and *SlCoAOMT4* in disease resistance, we co‐expressed the four BGC2 genes in *N. benthamiana*, using *SlEPS1* as a positive control, and then inoculated plants with *Pst DC3000* (Figure S10A, B). When *SlCoAOMT2*, *SlCoAOMT3*, or *SlCoAOMT4* was expressed individually, the *N. benthamiana* leaves showed enhanced disease resistance (Figure S10C–H). Co‐expression of *SlCoAOMT2‐SlCoAOMT3‐SlCoAOMT4* resulted in even stronger resistance, with significantly lower bacterial growth (Figure S10I, J). When all four genes were co‐expressed, *N. benthamiana* resistance to *Pst DC3000* was significantly improved, with a reduction in bacterial growth of up to 15% (Figure S10K, L). Furthermore, after transiently expressing *SlCoAOMT2*, *SlCoAOMT3*, and *SlCoAOMT4* in *N. benthamiana*, respectively, we observed a significant increase in the levels of Fer‐Spd and diFer‐Spd compared to the control (Figure S10M, N). We then generated *Slcoaomt4* mutant lines, named *Slcoaomt4‐KO*, using the CRISPR/CRISPR‐associated protein 9 (Cas9) system. Disease resistance evaluation showed that the *Slcoaomt4‐KO* lines were highly susceptible to *Pst DC3000* (Figure 4C), with significantly higher bacterial growth compared to WT plants (Figure 4D). Metabolomic analyses further indicated that the levels of Fer‐Spd and diFer‐Spd were significantly reduced in the *Slcoaomt4‐KO* lines (Figure 4E). Moreover, analysis of transcriptomic data from plants infected with eight different pathogens revealed that BGC2 gene expression was induced by infection, suggesting a potentially more general role in pathogen defense (Figure S11A–H) (Jeon et al., 2020). Following infection with *Ralstonia solanacearum* and *Xanthomonas euvesicatoria*, *SlEPS1* expression was upregulated by approximately 500 to 1,200 times compared to that in control plants (Figure S11I, J). These findings provide strong evidence that BGC2 is involved in phenolamide biosynthesis and confers disease resistance in tomato plants.

To comprehensively assess the evolutionary history of the BGC2–PBS3 module, we employed comparative genomics by examining 138 published genomes. The tomato BGC2–PBS3 module exhibited collinearity with corresponding genomic regions in species such as *Capsicum annuum*, *Manihot esculenta*, *Solanum melongena*, *Solanum pennellii*, *Solanum tuberosum*, and *Theobroma cacao* (Figure 4F). These regions were distributed across chromosomes CaChr.2, MeChr.13, SmChr.2, SpChr.2, StChr.2, and TcChr.1, respectively (Figure 4G). Further analysis revealed that the genomes of these seven species have undergone varying degrees of chromosomal rearrangements and tandem gene duplication events (Figure 4H). In the Solanaceae species, for instance, each genome showed evidence of a single chromosomal rearrangement and tandem duplication event, resulting in the inversion of the BGC2–PBS3 module (Figure 4H). Based on these data, we constructed a possible evolutionary history for the BGC2–PBS3 module, depicted on a species tree (Figure 4H).

### *SlMYB78* moderates BGC2 expression to enhance phenolamide and SA biosynthesis and improve plant disease resistance

**Figure 5:**
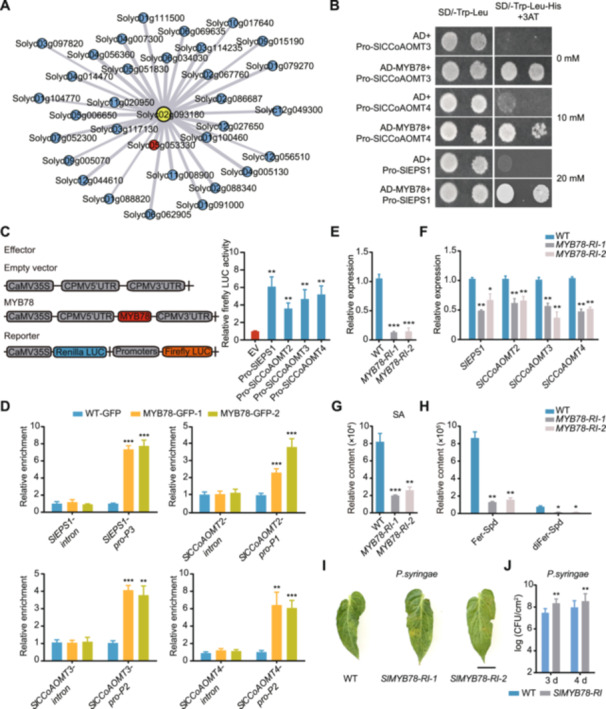
***SlMYB78*** **regulates expression of the biosynthetic gene cluster on chromosome 2 (BGC2) to enhance plant disease resistance** **(A)** Network illustrating correlations between *SlEPS1* and various transcription factors. *SlEPS1* is marked in yellow, and the most strongly correlated transcription factor is marked in red; all other transcription factors are marked in blue. **(B)** Yeast one‐hybrid assay of interactions between SlMYB78 and the promoters of genes within BGC2. The 3‐AT content for each assay is indicated on the right. Prefixes: 3‐AT, 3‐amino‐1,2,4‐triazole; AD, activation domain; Pro, promoter. **(C)** Transcription activation analysis of *SlMYB78* in *Nicotiana benthamiana* leaves. **(D)** Recruitment of SlMYB13 to the promoters of BGC2 *in vivo* by chromatin immunoprecipitation assays. **(E)** Expression analysis in *SlMYB78*‐silenced lines. **(F)** Relative expression of the BGC2 genes in *SlMYB78*‐silenced lines. **(G**, **H)** Heat map of the abundance of multiple phenolamides detected in *SlMYB78*‐silenced lines, with wild‐type (WT) plants serving as the control. The color scale indicates the log^2^ of the relative mass spectral intensity values. **(I**, **J)** *SlMYB78*‐silenced lines were more susceptible to *Pst DC3000* infection than WT plants **(I)**. Bacterial growth on inoculated tomato leaves was quantified at the depicted time points **(J)**. Inoculation was carried out using *Pseudomonas syringae* bacteria at 10^5^ colony‐forming units/mL (*n* = 12). All experiments were repeated twice with similar results. Data are shown as the mean ± *SD*, *n* = 3. *P < 0.05, **P < 0.01 and ***P < 0.001; Student's *t*‐tests.

To uncover the transcriptional regulatory network of *SlEPS1*, we used *SlEPS1* as the bait and performed a co‐expression analysis using published transcriptomic data (Li et al., 2020). The MYB transcription factor *Solyc05g055330* showed the most significant correlation with *SlEPS1* (Figure 5A; Table S5). A phylogenetic analysis demonstrated that the MYB protein encoded by *Solyc05g055330* grouped with AtMYB78 from *Arabidopsis thaliana* (Figure S12A), named SlMYB78. Subcellular localization analysis showed that SlMYB78 was localized in the nucleus (Figure S12B). Promoter analysis identified multiple MYB binding elements in each component of BGC2 (Figure S12C).

To confirm whether BGC2 is directly regulated by SlMYB78, we conducted yeast one‐hybrid assays (Y1H), which demonstrated that SlMYB78 specifically binds to the promoters of genes within BGC2 (Figure 5B). Electrophoretic mobility shift assays (EMSA) further confirmed the interaction between SlMYB78 and the predicted binding sites of the BGC2 promoters (Figure S12D–G). Dual‐luciferase reporter assays (LUC) revealed that *SlMYB78* strongly activated BGC2 promoters in *N. benthamiana* leaves (Figure 5C). Chromatin immunoprecipitation (ChIP) followed by qPCR targeting the MYB motifs in the BGC2 promoters further confirmed binding enrichment of SlMYB78 to these motifs (Figure 5D). These experimental approaches unequivocally established that SlMYB78 directly binds to and activates the promoters of genes in BGC2.

To further validate these findings, we generated *SlMYB78*‐silenced plants (*SlMYB78‐RNAi*) using RNA interference (RNAi) technology (Figure 5E), and observed a significant reduction in the expression of BGC2 component genes (Figure 5F). Compared to WT plants, the accumulation of SA, Fer‐Spd and diFer‐Spd was significantly reduced in *SlMYB78‐RNAi* plants (Figure 5G, H). After inoculation with *Pst DC3000*, the *SlMYB78‐RNAi* lines showed heightened pathogen sensitivity (Figure 5I), with significantly higher bacterial growth than in WT plants (Figure 5J). Similarly, *SlMYB78* expression was induced by infection with nine different pathogens (Figure S13). In summary, *SlMYB78* participated in phenolamide and SA biosynthesis by directly regulating the expression of BGC2 genes, thereby positively modulating plant resistance to *P. infestans*.

**Figure 6:**
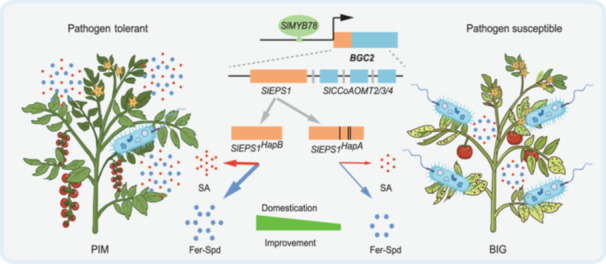
**A working model for the domestication‐linked gene cluster biosynthetic gene cluster on chromosome 2 (BGC2) and the transcription factor** ***SlMYB78*** **in mediating tomato disease resistance** The BGC2 gene cluster is responsible for the biosynthesis of spermidine (Spd)‐derived phenolamides and salicylic acid (SA). Acting as a positive regulator of this cluster, *SlMYB78* enhances phenolamide and SA production, thereby endowing tomato plants with disease resistance. *SlEPS1*, a pivotal gene within BGC2, possesses dual roles, facilitating the synthesis of both phenolamides and SA. The disease‐resistant *SlEPS1* ^HapB^ experienced negative selection during tomato domestication and improvement. Blue hexagons represent feruloyl (Fer)‐Spd, while red circles symbolize SA.

By integrating our findings, we propose a model of tomato disease resistance (Figure 6) where *SlMYB78* directly regulates the expression of BGC2, which is involved in phenolamide and SA biosynthesis. The core gene within BGC2, *SlEPS1*, has dual functions, catalyzing the synthesis of both phenolamides and SA. Notably, the elite haplotype *SlEPS1* ^HapB^, predominantly found in PIM tomato varieties, is relatively rare. During tomato domestication and improvement, *SlEPS1* ^HapB^ was negatively selected, leading to reductions in the phenolamide and SA content in modern cultivars and thereby negatively affecting disease resistance.

## DISCUSSION

In the field of biochemistry, the multifunctionality of enzymes is fundamental to their roles in complex metabolic pathways. Enzymes, including acyltransferases, glycosyltransferases, and methyltransferases, exhibit dual or multifunctional characteristics by interacting with a wide range of substrates. This versatility not only enhances the adaptability of enzymes within metabolic pathways but also provides a dynamic mechanism for cells to adapt to environmental changes (Dai et al., 2020; Wang et al., 2021). For instance, the VTC4 protein in plants demonstrates bifunctional properties, contributing to both the ascorbic acid and inositol metabolic pathways (Torabinejad et al., 2009). LPCAT3 is known to possess acyltransferase, lysophosphatidylcholine, lysophosphatidylethanolamine, and lysophosphatidylserine activities, facilitating the incorporation of polyunsaturated acyl chains into various membrane phospholipids (Zhang et al., 2021). Moreover, the flavonoid acyltransferase AtPMT1 exhibits a wide substrate spectrum and can act on nine acyl receptors, including daidzin and kaempferol 3/7‐*O*‐glucoside (Taguchi et al., 2010). OsGSA1 exhibits broad‐spectrum glycosyltransferase activity, regulating the flavonoid glycoside profile and lignin content in rice (Dong et al., 2020). In this study, we discovered that the acyltransferase SlEPS1 not only catalyzes SA synthesis but also phenolamide formation, significantly expanding our knowledge of acyltransferases function. The catalytic center of SlEPS1 possesses multiple binding sites enabling adaptation to different substrates and aiding in the efficient metabolic regulation and rapid synthesis of defensive compounds in response to environmental changes (Xu et al., 2021; Wang et al., 2023). Comparative analysis with AtEPS1 revealed conserved amino acid residues critical for substrate binding and catalysis (Torrens‐Spence et al., 2024), indicating similar functions. However, a key residue, His169 in SlEPS1, located in the BAHD family active site HXXXD, differed from that in AtEPS1 (Ser160 in AtEPS1). Site‐directed mutagenesis confirmed that altering His169 to Ser169 in SlEPS1 drastically reduces phenolamide catalytic activity, highlighting the importance of this residue for dual functionality of SlEPS1. Overall, this study deepens our understanding of acyltransferase functioning and offers novel insights for future research of metabolite regulation and biosynthesis.

Recent research has underscored the importance of phenolamides as biomarkers in plant defense pathways, as they undergo dynamic changes during pathogen infection and resistance induction (Zeiss et al., 2020). For example, phenolamides derived from putrescine and tyramine are critical biomarkers of *P. infestans* and *Pseudomonas syringae* pathogenicity (Doddaraju et al., 2013; Campos et al., 2014). Our study revealed that overexpression of *SlEPS1* in tomato significantly increased Fer‐Spd accumulation, enhancing resistance to *Pst DC3000*. Phenolamides also function as natural antimicrobials, with barley producing a novel phenolamide, *p*‐coumaroyl‐hydroxyputrescine, which exhibits antifungal activity in response to powdery mildew infection (von Roepenack‐Lahaye et al., 1998). *In vitro* antibacterial studies have confirmed that phenolamides directly inhibit *Pst DC3000* infection. In addition, phenolamide metabolism is intricately linked to plant signaling pathways in response to environmental stress. Positive feedbacks among abscisic acid (ABA), phenolamide, and polyamine regulatory pathways promote the biosynthesis of these three compounds and facilitate plant stress resistance (Urano et al., 2009; Cuevas et al., 2014; Cao et al., 2024). Salicylic acid, a key hormone involved in plant defense (Peng et al., 2021), regulates polyamine synthesis and catabolism, while polyamines increase SA levels in response to abiotic and biotic stresses (Iqbal et al., 2006; Radojičić et al., 2018; Zhang and Zhang, 2022). For instance, polyamine accumulation in *npr1‐1* and *mpk6‐2* plants affects the SA response to *P. syringae* infection; the activation of polyamines post‐infection triggers local SA accumulation and systemic transcriptional reprogramming, activating SA‐dependent defense responses (Liu et al., 2020; Rossi et al., 2021). Our study found that phenolamides and SA mutually influence each other's synthesis and work synergistically to enhance disease resistance in tomato. However, the interaction between phenolamides and SA still requires further investigation.

Phenolamide gene clusters often play a pivotal role in the synthesis of secondary metabolites, particularly in plant responses to abiotic and biotic stresses (Zhan et al., 2022). For example, in rice, two such clusters are responsible for the synthesis of phenolamides from putrescine and tyramine, contributing to resistance against pathogenic bacteria (Fang et al., 2021; Shen et al., 2021). In tomatoes, putrescine‐ and spermidine‐derived phenolamide biosynthetic gene clusters are involved in drought stress resistance (Cao et al., 2024). In this study, we identified and characterized a gene cluster, BGC2, comprising multiple enzyme‐coding genes that collectively contribute to phenolamide biosynthesis. We found that the expression of BGC2 is induced by various pathogens and plays a crucial role in plant stress resistance. Overexpression of *SlEPS1*, a core gene within BGC2, significantly increased phenolamide accumulation, thereby enhancing disease resistance. In contrast, *Sleps1‐KO* lines were more sensitive to *Pst DC3000* infection. Transient overexpression of the BGC2 genes in *N. benthamiana* leaves also significantly improved resistance to *Pst DC3000*, suggesting an additive effect of the cluster genes. Secondary metabolic gene clusters are typically regulated by transcription factors (TFs) and expressed in specific tissues and/or under specific stresses (Takos and Rook, 2012; Töpfer et al., 2017; Zhan et al., 2022). For instance, the bZIP transcription factor *APIP5* negatively regulates the transcription of *OsPHT4*, a core gene within the phenolamide gene cluster, reducing resistance to *M. oryzae* (Fang et al., 2021). The MYB transcription factor *SlMYB13* targets and positively regulates the phenolamide gene clusters BGC7 and BGC11, promoting the efficient coordination of phenolamide metabolic pathways and enhancing tomato drought tolerance (Cao et al., 2024). In this study, we found that *SlMYB78* expression is induced by various pathogens and activates the expression of the downstream phenolamide gene cluster BGC2, conferring disease resistance in tomato plants. In addition, natural variation plays a significant role in the functional regulation of phenolamide gene clusters. We compared the disease resistance of *SlEPS1* ^HapB^ plants (introduced from the wild variety TS‐21) with that of *SlEPS1* ^HapA^ plants (introduced from the modern cultivar TS‐108) in a MicroTom tomato background. Plants carrying the *SlEPS1* ^HapB^ haplotype exhibited significantly higher pathogen resistance compared to those carrying the *SlEPS1* ^HapA^ haplotype. Therefore, by identifying and integrating beneficial haplotypes with key gene clusters, we expect to develop tomato varieties with enhanced stress resistance, addressing challenges posed by climate change and improving tomato quality and yields, thereby bringing greater economic benefits to agricultural production.

## MATERIALS AND METHODS

### Plant materials and metabolite sample preparation

In this study, we utilized a global diversity population comprising 401 accessions of tomato (*Solanum lycopersicum*), covering multiple lines such as PIM, CER, and BIG. Table S1 provides detailed information on these 401 accessions, including variety names, countries of origin, geographic coordinates, and subpopulation classifications. These materials were planted in Changjiang, Hainan, according to a randomized complete block design. Each strain was planted in two rows, six plants per row, and with two replicates. Leaf samples were collected at the four‐leaf stage for metabolomic analysis. Field management, such as irrigation, fertilization, and pest control, strictly followed traditional agricultural standard operating procedures.

We cloned the full‐length CDS of various genes from complementary DNA (cDNA) prepared from mature tomato leaves using primers provided in Table S7. The purified PCR products were inserted into the intermediate vector pDONR207, and the correct sequence verified by sequencing was transferred into the modified pBI121 vector by LR reaction, which carried the CaMV35S promoter. The vector was then transformed into *Agrobacterium tumefaciens* LBA4404 and subsequently transferred into MicroTOM, employing a modified protocol that was based on a described method (Ying et al., 2020). Two independent T1 homozygous lines were used in this study. The gene expression levels of these lines were determined by relative quantitative qRT‐PCR. For each transgenic line, at least two independent lines were selected for targeted metabolite analysis.

Leaf samples were collected from 4‐week‐old seedlings and quickly frozen in liquid nitrogen. The extraction of tomato samples followed established methods. Briefly, freeze‐dried samples were ground at 30 Hz for 1 min using a Ratsch MM 400 mixer mill with zirconia beads; then, 100 mg of dried powder was extracted in 1.0 mL of 70% aqueous methanol containing 0.1 mg/L lidocaine (internal standard) and incubated overnight at 4°C, followed by centrifugation at 10,000 *g* for 10 min. The supernatant was filtered through a 0.22 μm SCAA‐104 filter (ANPEL, Shanghai, China) before liquid chromatography – electrospray ionization – tandem mass spectrometry (LC‐ESI–MS/MS) analysis (Zhu et al., 2018).

### Phenolamide and SA content detection in tomato

For metabolome analysis, samples were analyzed using a low‐resolution AB SCIEX 6500 QTRAP LC/MS. Separation of metabolites was performed on a C18 column (2.1 × 100 mm, 1.9 µm, shim‐pack VP‐OSD). The mobile phase consisted of 0.04% acetic acid in water (phase A) and 0.04% acetic acid in acetonitrile (phase B). The analysis was carried out with an elution gradient as follows: 0 min, 5% B; 0–10 min, 5%–95% B; 10–11 min, 95% B; 11–11.1 min, 95%–5% B; 11.1–14 min, 5% B; the injection volume was 2 µL; flow rate was 0.35 mL/min; and the temperature was 40°C. The ESI source conditions were set as follows: the source temperature was 500°C; the ion spray voltage was 5,500 V; the ion source gas (GSI), gas II (GSII), and current (CUR) were set at 55, 60, and 25 psi, respectively, and the collision‐activated dissociation (CAD) was set to high mode. Each sample was performed in the scheduled multiple reaction monitoring (s‐MRM) mode by 6500 QTRAP using Multi Quant 3.0.3 software for the quantification of metabolites. The detection scan window was set to 60 s, and the target scan time was 1.5 s. In order to improve the normalization, the relative signal strength of the metabolite was divided and normalized according to the internal standards (0.1 mg/L lidocaine), and log^2^ was then used to transform the value (Table S2).

### Genome‐wide association analysis

A GWAS was performed using 2,318,614 SNPs (minor allele frequency (MAF) > 5% and missing rate < 10%) in the leaves of 401 tomato lines. Association analysis was performed using the efficient mixed model software Expedited (EMMAX) (Kang et al., 2010). In this analysis, the pairwise genetic distance matrix was used as the variance‐covariance matrix for random effects, while the top 10 principal components calculated by the GCTA software were included as fixed effects. A uniform significance threshold was set for all traits across the genome (*P* = 1/*n*, where *n* is the number of independent SNPs). The number of independent SNPs was determined using the Genotype Error Calculator (GEC) software (Li et al., 2012). For all metabolite traits, a uniform significance threshold (*P* = 5.1 × 10^−5^) was used to screen the SNPs.

### Phylogenetic analysis

In this study, we extracted tomato protein sequences from the SGN database (website: https://solgenomics.net/). Additionally, we obtained acyltransferase, methyltransferase, and MYB protein sequences from other plant species from the National Center for Biotechnology Information (NCBI: http://www.ncbi.nlm.nih.gov/) and Phytozome 13 (https://phytozome-next.jgi.doe.gov/) databases. Amino acid sequence alignments were performed using ClustalW software, with results detailed in Table S3. On this basis, we constructed a neighbor‐joining phylogenetic tree using the default parameters of the MEGA6 software (Temple University, Philadelphia, PA, USA) with default parameters. The reliability of the branches within the phylogenetic tree was assessed using a Bootstrap test with 1,000 replicates.

### Recombinant protein expression and enzyme activity assays

The verified pDONR207 vector was cloned into the pGEX‐6p‐1 expression vector (Novagen, Madison, WI, USA), carrying a glutathione S‐transferase (GST) tag, via LR recombination technology (Invitrogen, Carlsbad, CA, USA). Recombinant protein analysis and enzyme activity assays were performed with slight modifications to the reported method (Peng et al., 2017). Induced expression of recombinant proteins was achieved by culturing at 18°C with 0.15 mmol/L isopropyl‐β‐D‐thiogalactopyranoside (IPTG) for 16 h.

To characterize the biochemical function of EPS1 in catalyzing SA, we produced isochorismoyl‐glutamate conjugates enzymatically in 100‐mL reaction systems containing 50 mmol/L Tris, 1 mmol/L L‐glutamate, 5 mmol/L chorismate, 5 mmol/L ATP, and 5 mmol/L MgCl_2_ with addition of 5 mg of ICS1 and 5 mg of PBS3, and incubated them for 20 min at 25°C. Next, EPS1 were added to the reactions and incubated for an additional 5 min at 25°C prior to quenching with an equal volume of MeOH (Torrens‐Spence et al., 2019). To characterize the biochemical function of EPS1 in catalyzing phenolamides, an experiment was conducted in a 100 μL reaction system containing 1 mmol/L spermidine substrate, 0.2 mmol/L Fer‐CoA donor, 2.5 mmol/L MgCl_2_, and 100 mmol/L Tris‐HCl buffer (pH 7.5) with 500 ng of purified protein. The mixture was incubated at 37°C for 40 min.

*In vitro* activity assays for methyltransferase were similarly performed in a 100 μL reaction system containing 500 ng of purified protein, 0.2 mmol/L SAM methyl donor, 1 mmol/L Fer‐CoA substrate, 2.5 mmol/L MgCl_2_, and 100 mmol/L Tris‐HCl buffer (pH 7.5). The mixture was incubated at 30°C for 1 h. Reactions were terminated by adding 500 μL of ice‐cold methanol, and the supernatant was filtered before analysis by LC–MS. Quantitative analysis of phenolamides was performed using standards for calibration. *In vitro* experimental results were judged by comparison with retention times and MS/MS spectra of the standards.

### Heterologous expression of candidate proteins in *N. benthamiana*

Using LR recombination technology (Invitrogen, Carlsbad, CA, USA), the sequencing‐confirmed clone (vector pDONR207) was cloned into the pEAQ‐HT‐DEST2 vector (Sainsbury et al., 2009). The pEAQ‐HT plasmids containing either the candidate tomato gene or green fluorescent protein (GFP, as a negative control) were then transformed into *A. tumefaciens* (GV3101) and cultured on Luria–Bertani (LB) plates containing 50 mg/mL kanamycin and 30 mg/mL gentamicin at 30°C for 3 d. After that, the transformed *A. tumefaciens* were cultured in LB medium containing 50 μg/mL kanamycin and washed with washing buffer (10 mmol/L 2‐(N‐morpholino) ethanesulfonic acid (MES), pH 5.6), and resuspended in MMA buffer (10 mmol/L MES, pH 5.6; 10 mmol/L MgCl_2_; 100 mmol/L acetosyringone) to an optical density at 600 nm (OD_600_) = 1.0. After incubation at room temperature for 2 h, 1 mL of the bacterial suspension was infiltrated into the abaxial side of the leaves of 6‐week‐old *N. benthamiana* using a needleless 1 mL syringe. Leaves from different plants (*n* = 3) were harvested 3 d post‐infiltration (dpi), rapidly frozen in liquid nitrogen, and stored at −80°C. Tobacco sample extraction followed the same method as the aforementioned metabolite analysis.

### CRISPR/Cas9‐mediated gene editing

The CRISPR/Cas9 target sites were selected using the CRISPR‐P 2.0 online tool (http://cbi.hzau.edu.cn/CRISPR2/). Specifically, target sites for each gene were selected and designed into the sequences of forward and reverse PCR primers. Fragments were amplified from the p43 vector by PCR, purified, and then cloned into the *Bsa* I site of the pTX041 vector using the Golden‐Gate assembly method (Sun et al., 2020). The correctness of the construction was verified by restriction enzyme digestion analysis and DNA sequencing. The constructed plasmids were then transformed into *A. tumefaciens* LBA4404 strain and transferred into MicroTOM plants according to the previously described method (Du et al., 2014). CRISPR/Cas9‐induced mutations were genotyped by PCR amplification and DNA sequencing. Primer sequences are listed in Table S7.

### RNA extraction and gene expression analysis

Total RNA was extracted from tomato leaves using TRIzol reagent (Invitrogen, Carlsbad, CA, USA) following the manufacturer's protocol. In a 20 μL reaction system, 3 μg of RNA was added, and first‐strand cDNAs were prepared using the EasyScript One‐Step gDNA Removal and cDNA Synthesis SuperMix from TransGen (Beijing, China). Transcript abundance was quantified using TB GreenTM Premix Ex TaqTM II (Tli RNaseH Plus) or SYBR Green PCR Master Mix (RR420A, TaKaRa) on an ABI 7500 Real‐Time PCR System (Applied Biosystems, Foster City, CA, USA). The expression level of each sample was determined using the relative quantification method proposed by Livak and Schmittgen and normalized to the tomato actin gene (Livak and Schmittgen, 2001). The primer sequences for qRT‐PCR are detailed in Table S7.

### Pathogen inoculation and quantification of bacterial growth

Pathogen inoculation was performed with adjustments to previously published protocols (Hu et al., 2022). The *Pst DC3000* strain was inoculated on King's B solid medium containing 25 mg/L rifampicin and cultured at 28°C for 2 d. A single colony was selected and inoculated into King's B liquid medium containing 25 mg/L rifampicin, cultured at 28°C, 200 rpm for 8 to 12 h. After that, it was centrifuged at 4,000 *g* for 5 min at 4°C, the supernatant was discarded. It was washed twice with 10 mmol/L MgCl_2_ solution and resuspended, the bacterial concentration was adjusted to OD_600_ = 0.05. The bacterial suspension containing 0.03% Silwet L‐77 was uniformly sprayed on tomato leaves as a treatment group, while the control group was sprayed with a 10 mmol/L MgCl_2_ solution containing 0.03% Silwet L‐77. Tissue samples were collected and total RNA was extracted for qRT‐PCR analysis at 0, 2, 4, 8, 12, 24, and 48 h after inoculation with *Pst DC3000*. The experiment was independently repeated at least three times, and similar results were obtained each time.

Quantification of bacterial growth was performed with adjustments to previously published protocols (Wu et al., 2019). At 3 and 4 d after inoculation with *Pst DC3000*, fully expanded functional leaves were sampled randomly using a hole punch, then sterilized in 70% ethanol for 10 s, washed twice with sterile water, and placed in 500 μL of 10 mmol/L MgCl_2_ solution for thorough grinding. Three biological replicates were set for each treatment. The grinding liquid was serially diluted 10^−1^, 10^−2^, 10^−3^, 10^−4^, 10^−5^, and 10 μL of the original liquid and dilutions were spotted onto King's B solid medium containing 25 mg/L rifampicin. The plates were cultured at 28°C for 2 d. Each dilution concentration had three replicates.

### *In vitro* evaluation of antimicrobial activity

Liquid antimicrobial assay was performed with adjustments to previously published protocols (Su et al., 2024). A single colony was selected from R2A agar plates and cultured overnight in R2A medium at 180 rpm, 28°C. The cultures were centrifuged at 600 *g* for 10 min at 4°C to collect bacteria. The collected bacteria were resuspended in R2A medium to OD_600_ = 0.01, and then sterile 1 mol/L SA or Fer‐Spd solution (dissolved in ethanol and filtered through a 0.22 μm filter) was added to achieve different concentrations. As a control, an equal volume of ethanol solvent (filtered through a 0.22 μm filter) was added. The effects of SA or Fer‐Spd on the growth of *Pst DC3000* were evaluated by using R2A medium supplemented with different concentrations of SA or Fer‐Spd.

Solid antimicrobial assay was performed with adjustments to previously published protocols (Liang et al., 2022). A suitable amount of bacterial suspension was accurately pipetted using a liquid culture medium to prepare the required concentration. Pre‐sterilized agar medium was heated until fully dissolved and evenly poured into Petri dishes, with 15 mL per dish as the base layer, and allowed to solidify. After the medium cooled to around 50°C, experimental bacterial strains were added, mixed thoroughly, and 5 mL of the bacterial‐mixed medium was spread over the solidified base layer to form the upper layer, then allowed to solidify. Oxford cups were placed vertically on the medium surface, lightly pressed to ensure close contact with the medium without leaving gaps. Different concentrations of SA or Fer‐Spd solution were added to the Oxford cups, with 200 μL per cup, ensuring no overflow. After adding the solution, these were incubated at 37°C for 16–18 h, followed by observation of the results.

### Subcellular localization assay

The CDS of SlMYB78 was cloned into the pH7WGF2‐GFP vector and expressed in fusion with the GFP protein under the control of the CaMV 35S promoter. Colocalization analysis was performed using the 35S:PIF4n‐OFP marker with a nuclear localization signal (Batistič et al., 2010). The recombinant vector was introduced into *A. tumefaciens* GV3101 and infiltrated into 4‐week‐old *N. benthamiana* leaves. Co‐focused images were obtained using a laser scanning confocal microscope (Leica TCS SP5).

### Yeast one‐hybrid assay

The full‐length cDNA sequence of *SlMYB78* was amplified and fused to the GAL4 activation domain in the pGADT7 vector (Clontech, Shiga, Japan) to construct the pGADT7‐*SlMYB78* recombinant plasmid. Meanwhile, 2‐kilobase pair (kb) promoter regions of *SlEPS1*, *SlCCoAOMT2*, *SlCCoAOMT3*, and *SlCCoAOMT4* were amplified and cloned into the pHIS2 reporter vector. The pHIS2 bait vector containing the target gene promoter and the pGADT7‐*SlMYB78* prey vector were co‐transformed into yeast cells. As a negative control, yeast cells were transformed with the empty pGADT7 vector and the corresponding promoter‐containing pHIS2 vector. Transformed yeast clones were grown on synthetic dropout (SD)‐Leu‐Trp medium and SD‐Leu‐Trp‐His medium supplemented with 3‐AT (Sigma, St. Louis, MO, USA) at 30°C for 3 d. Relevant cloning primers and promoter information are detailed in Table S7.

### Electrophoretic mobility shift assay

Recombinant SlMYB78‐MBP protein was prepared and purified, and EMSA was performed with adjustments to previously published protocols (Shang et al., 2014). Double‐stranded promoter fragment probes (labeled with FAMA) for EMSA were obtained by PCR amplification, with primers listed in Table S7. The binding reaction was performed in a 10 μL reaction system containing binding buffer (1 mol/L Tris‐HCl, pH 8.0; 5 mol/L NaCl; 1 mol/L MgCl_2_; 10 mg/mL bovine serum albumin (BSA); 100% glycerol; 1 mol/L dithiothreitol (DTT)), 2 μL of fusion protein, and 2 μL of FAMA‐labeled promoter fragment, incubated at 25°C for 30 min in the dark. The samples after the reaction were separated on a 4% polyacrylamide gel and imaged on an ODYSSEY FC imaging system.

### Transactivation assay in tobacco leaves

The cDNA of *SlMYB78* was inserted into the pEAQ‐HT‐DEST2 vector, serving as the effector. Simultaneously, the 2‐kb promoter regions of *SlEPS1*, *SlCCoAOMT2*, *SlCCoAOMT3*, and *SlCCoAOMT4* were amplified and introduced into the modified pH2GW7 vector, which contained firefly luciferase (fLUC) and renilla luciferase (rLUC) as reporter genes. These plasmids were then transformed into *A. tumefaciens* GV3101, respectively. For co‐transformation experiments, bacterial cultures of equal volume and concentration were mixed and infiltrated into fully expanded leaves of 4‐week‐old *N. benthamiana* using a needleless syringe. After the infiltration treatment, the plants were incubated in the dark for 12 h, then grown under a 16‐h light/8‐h dark cycle for 48 h. Luciferase and rLUC activities were measured according to the manufacturer's instructions (Promega, Madison, WI, USA), with three independent replicates performed. Cloning primers and promoter details can be found in Table S7.

### Chromatin immunoprecipitation assay

Four‐week‐old WT and 35S:SlMYB78‐GFP transgenic seedlings were harvested and subjected to cross‐linking with a 1% formaldehyde solution under vacuum for 30 min. The plants were then frozen and ground into a fine powder in liquid nitrogen. The subsequent procedures were performed as previously described with minor modifications (Du et al., 2017). For immunoprecipitation, an agarose‐conjugated anti‐GFP antibody (Abcam Ab290) was utilized. The precipitated DNA fragments were analyzed using qRT‐PCR with the primer sequences (Table S7). Each ChIP result was normalized to the corresponding input DNA. Fold enrichment of the target promoters was determined relative to the *ACTIN2* promoter. PCR reactions were conducted in triplicate for each sample, and the mean value of the technical replicates was recorded for each biological replicate.

### Genomic synteny analysis across plant species

A homology search strategy was employed to identify genes homologous to the BGC2 module across multiple plant species. Genome sequences and corresponding gene annotation data for 138 plant species were downloaded from the Ensembl Plants (http://plants.ensembl.org/index.html), NCBI (https://www.ncbi.nlm.nih.gov/), Phytozome (https://phytozome-next.jgi.doe.gov/), and SGN (https://solgenomics.net/) databases. Homologous genes across different species were identified using the DIAMOND software (Buchfink et al., 2021). Genomic synteny relationships were analyzed using JCVI software (Tang et al., 2008). Table S6 lists the relevant information of the most similar homologous genes identified in 138 species with evolutionary representation to the BGC2 module.

## CONFLICTS OF INTEREST

The authors declare no conflict of interest.

## AUTHOR CONTRIBUTIONS

S.W. and Jun.Y. conceived and designed the research. P.C., L.X., XG.L., D.M., Z.W., and XY.L. participated in the material preparations. S.W., Z.Z., and J.L. conducted the metabolic profiling. P.C., Jun.Y., L.X., D.M., XG.L., Z.W., XY.L., Y.H., P.L., and Jie.Y. performed the experiments. P.C., C.W., and C.L. analyzed the data. P.C., Jun.Y., and S.W. wrote and revised the manuscript with input from all authors. All authors read and approved the contents of this paper.

## Supporting information

Additional Supporting Information may be found online in the supporting information tab for this article: http://onlinelibrary.wiley.com/doi/10.1111/jipb.13899/suppinfo

**Figure S1.** Heatmap of all phenolamides showing differential accumulation among the three tomato subpopulations
**Figure S2.** Phylogenetic analysis of acyltransferase and methyltransferases
**Figure S3.** Phylogenetic analysis and multiple sequence alignment of the EPS1 protein
**Figure S4.** Analysis of disease resistance and bacterial growth in *Nicotiana benthamiana* transiently expressing *SlEPS1* and *SlEPS1‐mut*
**Figure S5.** Analysis of the synergistic regulation of disease resistance by salicylic acid (SA) and phenolamides
**Figure S6.** Assessment of disease resistance of different tomato varieties among populations
**Figure S7.** Functional analysis of *SlEPS1* ^HapA^ *‐108* and *SlEPS1* ^HapB^ *‐21*
**Figure S8.** Co‐expression network map of biosynthetic gene cluster on chromosome 2 (BGC2) components and synthesis genes of salicylic acid (SA) and phenylamide
**Figure S9.** Upregulation of the biosynthetic gene cluster on chromosome 2 (BGC2) after inoculation
**Figure S10.** Analysis of antimicrobial defense and bacterial colonization patterns in *Nicotiana benthamiana* leaves with transient co‐expression of biosynthetic gene cluster on chromosome 2 (BGC2) components following *Pst DC3000* infection
**Figure S11.** Expression patterns of biosynthetic gene cluster on chromosome 2 (BGC2) component genes in tomato following induction by different pathogens
**Figure S12.** Analysis of the *SlMYB78* and biosynthetic gene cluster on chromosome 2 (BGC2)
**Figure S13.** Upregulation of the *SlMYB78* after inoculation

**Table S1.** Summary of the sampled collection of tomato
**Table S2.** Multiple reaction monitoring (MRM) transitions analysis of phenolamides in tomato.
**Table S3.** The protein sequences used for phylogenetic analyses
**Table S4.** Detailed information on the frequences of the two haplotypes
**Table S5.** Transcription factors identified by gene co‐expression analysis of *SlEPS1*
**Table S6.** Genomic locations of gene cluster members in different species
**Table S7.** Primers used in this study
