# Supplementary material for: A SlMYB78‐regulated bifunctional gene cluster for phenolamide and salicylic acid biosynthesis during tomato domestication, reducing disease resistance
Source: J Integr Plant Biol. 2025 Mar 28;67(7):1947–64. doi: 10.1111/jipb.13899 (PMC12225020; doi:10.1111/jipb.13899)
Supplement: Supplementary file 1 — Figure S1. Heatmap of all phenolamides showing differential accumulation among the three tomato subpopulations Figure S2. Phylogenetic analysis of acyltransferase and methyltransferases Figure S3. Phylogenetic analysis and multiple sequence alignment of the EPS1 protein Figure S4. Analysis of disease resistance and bacterial growth in Nicotiana benthamiana transiently expressing SlEPS1 and SlEPS1‐mut Figure S5. Analysis of the synergistic regulation of disease resistance by salicylic acid (SA) and phenolamides Figure S6. Assessment of disease resistance of different tomato varieties among populations Figure S7. Functional analysis of SlEPS1 HapA ‐108 and SlEPS1 HapB ‐21 Figure S8. Co‐expression network map of biosynthetic gene cluster on chromosome 2 (BGC2) components and synthesis genes of salicylic acid (SA) and phenylamide Figure S9. Upregulation of the biosynthetic gene cluster on chromosome 2 (BGC2) after inoculation Figure S10. Analysis of antimicrobial defense and bacterial colonization patterns in Nicotiana benthamiana leaves with transient co‐expression of biosynthetic gene cluster on chromosome 2 (BGC2) components following Pst DC3000 infection Figure S11. Expression patterns of biosynthetic gene cluster on chromosome 2 (BGC2) component genes in tomato following induction by different pathogens Figure S12. Analysis of the SlMYB78 and biosynthetic gene cluster on chromosome 2 (BGC2) Figure S13. Upregulation of the SlMYB78 after inoculation [file JIPB-67-1947-s002.docx]

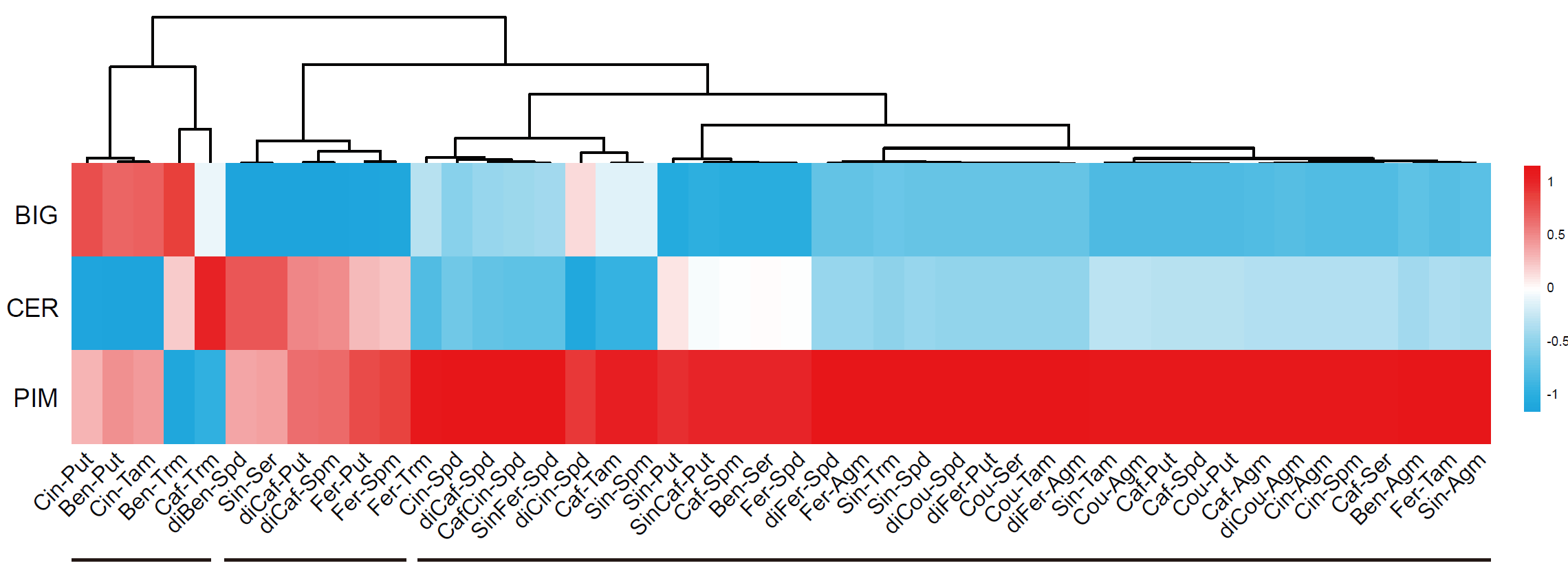


**Figure S1. Heatmap of all phenolamides showing differential accumulation among the three tomato subpopulations.**

Agm, agmatine; Put, putrescine; Spd, spermidine; Spm, spermine; Tam, tyramine; Trm, tryptamine; Ser, serotonin; Ben, benzoyl; Cin, cinnamyl; Cou, coumaroyl; Caf, caffeoyl; Fer, feruloyl; Sin, sinapoyl.


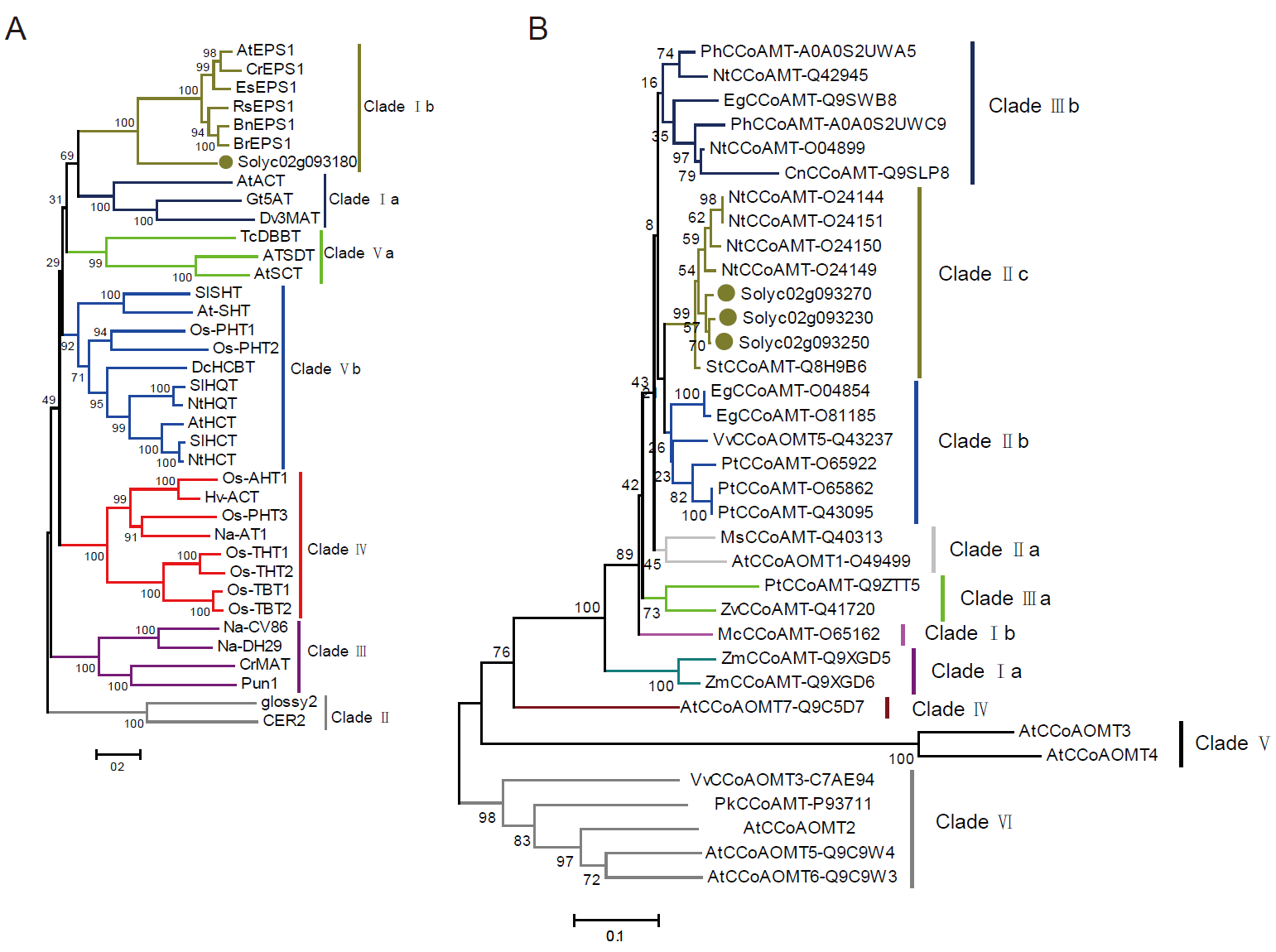


**Figure S2. Phylogenetic analysis of acyltransferase and methyltransferases.**

(**A**) Neighbor-joining tree of SlEPS1 and related acyltransferases. (**B**) Neighbor-joining tree of SlCoAOMT2, SlCoAOMT3, and SlCoAOMT4 and related methyltransferases. Phylogenetic analysis was performed by MEGA 7.0 using the neighbor-joining method and carried out via the bootstrapping method with 1000 replicates. At, *Arabidopsis thaliana*; Bn, *Brassica napus*; Br, *Brassica rapa*; Cn, *Citrus natsudaidai*; Cr, *Capsella rubella*; Dc, *Dianthus caryophyllus*; Eg, *Eucalyptus globulus*; Es, *Eutrema salsugineum*; Fe, *Fagopyrum esculentum*; Gm, *Glycine max*; Gt, *Gentiana triflora*; Hv, *Hordeum vulgare*; Mc, *Mesembryanthemum crystallinum*; Ms, *Medicago sativa*; Na, *Nicotiana attenuata*; Nt, *Nicotiana tabacum*; Os, *Oryza sativa*; Pl, *Pueraria lobata*; Pf, *Perilla frutescens*; Ph, *Petunia hybrida*; Pt, *Populus trichocarpa*; Pk, *Populuskita kamiensis*; Rs, *Raphanus sativus*; Sl, *Solanum lycopersicum*; St, *Solanum tuberosum*; Sb, *Scutellaria baicalensis*; Tc, *Taxus cuspidata*; Th, *Torenia hybrida*; Vv, *Vitis vinifera*; Zv, *Zinnia violacea*; Zm, *Zea mays*. The acyltransferase and methyltransferases amino acid sequences are presented in **Table** **S3**.


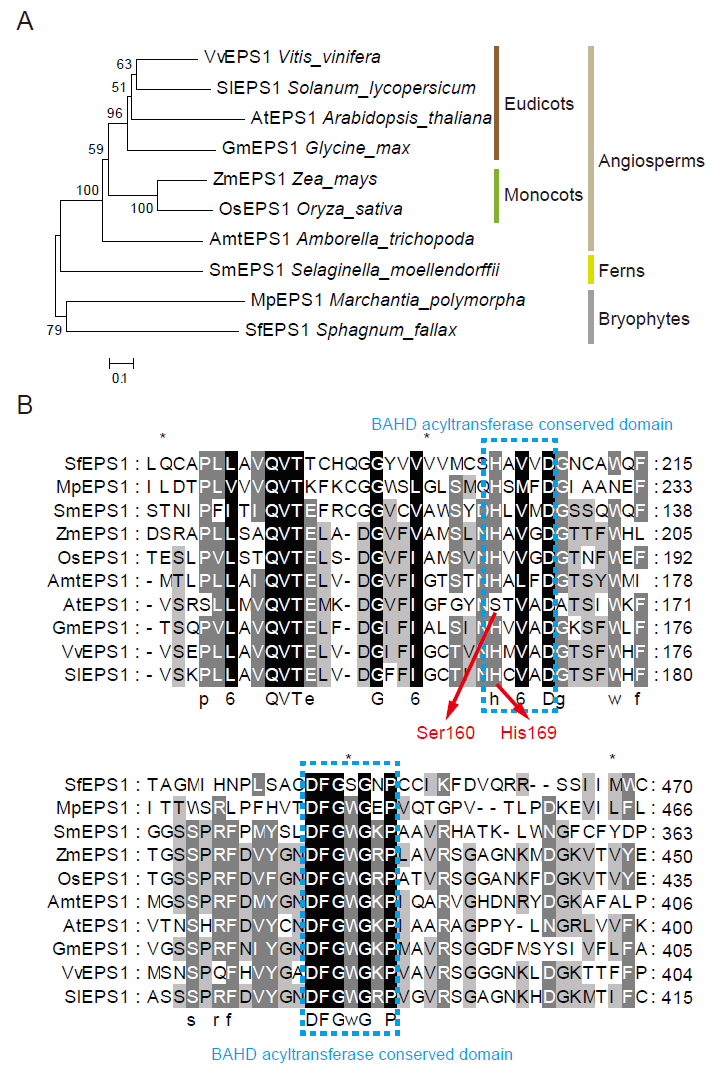


**Figure S3. Phylogenetic analysis and multiple sequence alignment of the EPS1 protein.**

(**A**) Evolutionary tree analysis of EPS1 proteins from different species. (**B**) Multiple sequence alignment of EPS1 proteins from different species, with the red arrow indicating the key amino acid residues within the catalytic site.


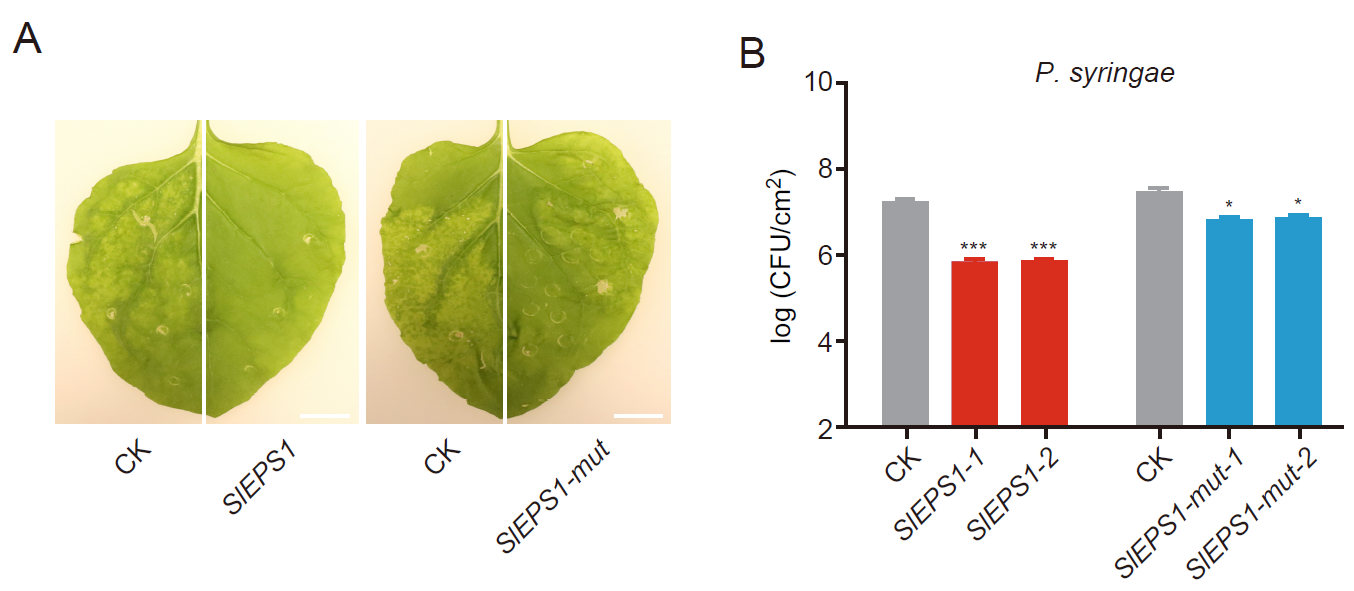


**Figure S4.** **Analysis of disease resistance and bacterial growth in *N. benthamiana* transiently expressing *SlEPS1* and *SlEPS1-mut*.**

(**A**) Evaluation of disease resistance in transiently expressed *SlEPS1* and *SlEPS1-mut* *N. benthamiana* leaves. Photographs were taken at 4 d after inoculation with *Pst DC3000*. d, day. Scale bar = 1 cm. (**B**) Comparisons of bacterial growth in transiently expressed *SlEPS1* and *SlEPS1-mut* *N. benthamiana* leaves at 4 d after inoculation.


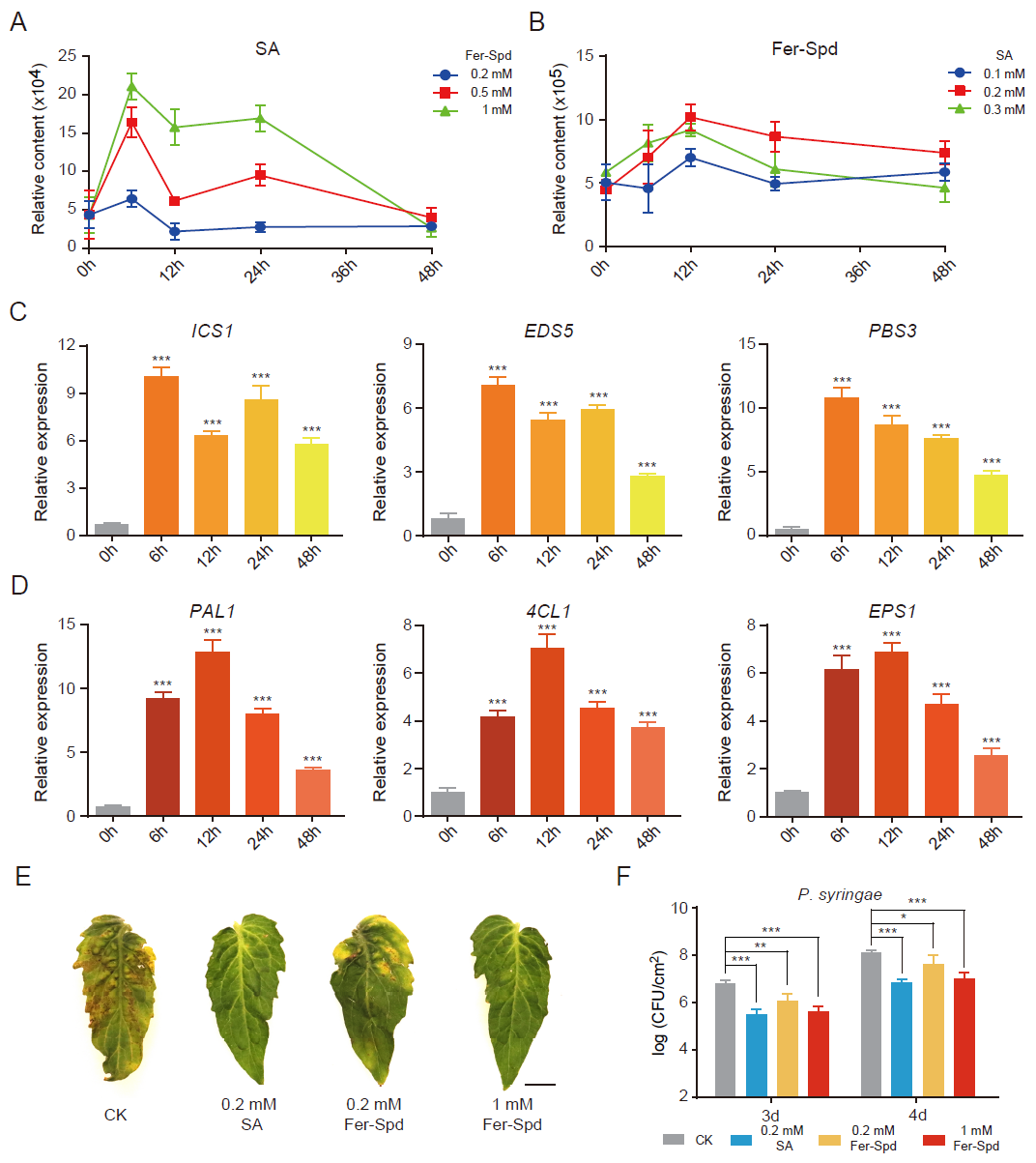


**Figure S5.** **Analysis of the synergistic regulation of disease resistance by SA and phenolamides.**

(**A**) Changes in SA levels at different time points in tomato leaves treated with various concentrations of Fer-Spd (0.2 mM, 0.5 mM, 1 mM). (**B**) Effects of different concentrations of SA (100 μM, 200 μM, 300 μM) on Fer-Spd concentrations at various time points in tomato leaves. (**C**) Impact of exogenous application of 1 mM Fer-Spd on the expression of SA biosynthesis genes (*ICS1*, *EDS5*, and *PBS3*) in tomato. (**D**) Impact of exogenous application of 0.2 mM SA on the expression of phenolamide biosynthesis genes (*PAL1*, *4CL1*, and *EPS1*) in tomato. (**E**) Evaluation of disease resistance in WT tomato plants after exogenous application of 200 μM SA, 200 μM Fer-Spd, and 1 mM Fer-Spd. Photographs were taken one week after inoculation with *Pst. DC3000*. Scale bar = 1 cm. WT, wild-type. (**F**) Quantification of *Pst. DC3000* growth in wild-type tomato plants after exogenous application of 200 μM SA, 200 μM Fer-Spd, and 1 mM Fer-Spd.


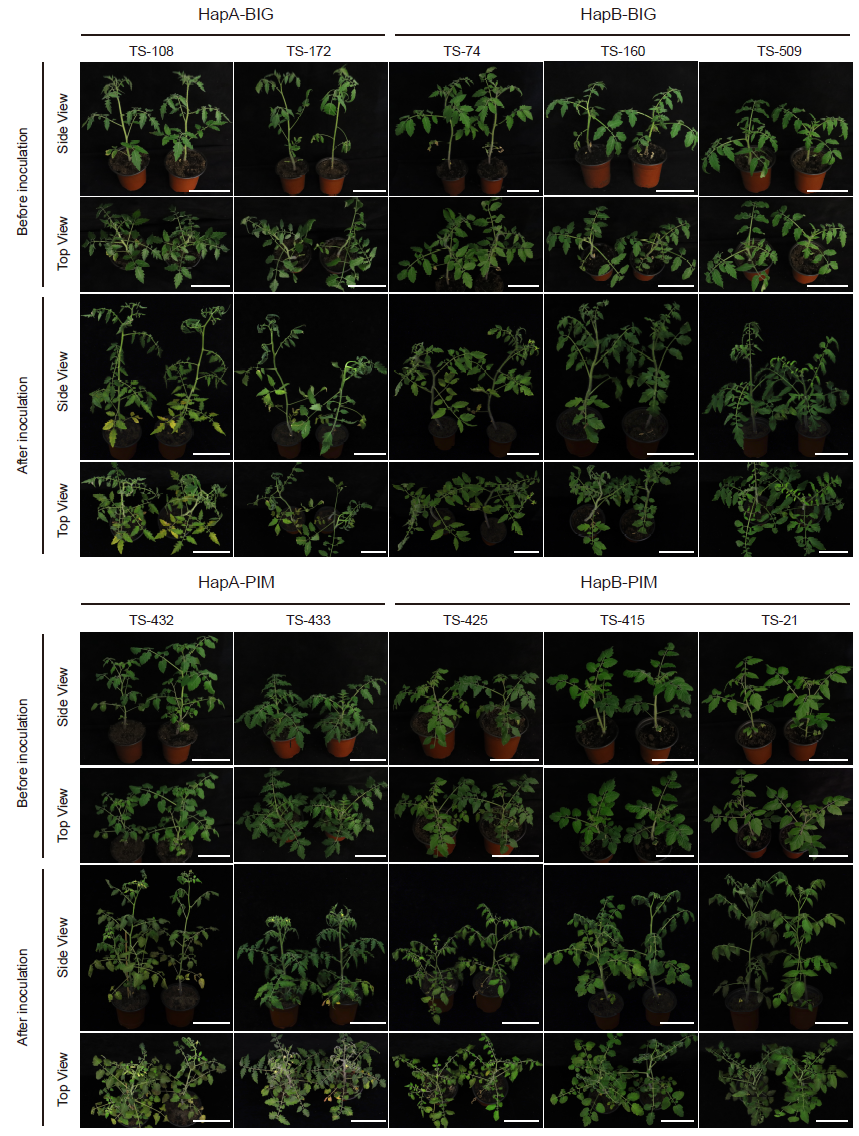


**Figure S6. Assessment of disease resistance of different tomato varieties among populations.**

Photographs were taken under normal conditions and at 7 d after inoculation with *Pst DC3000*. d, day. Scale bar =10 cm.


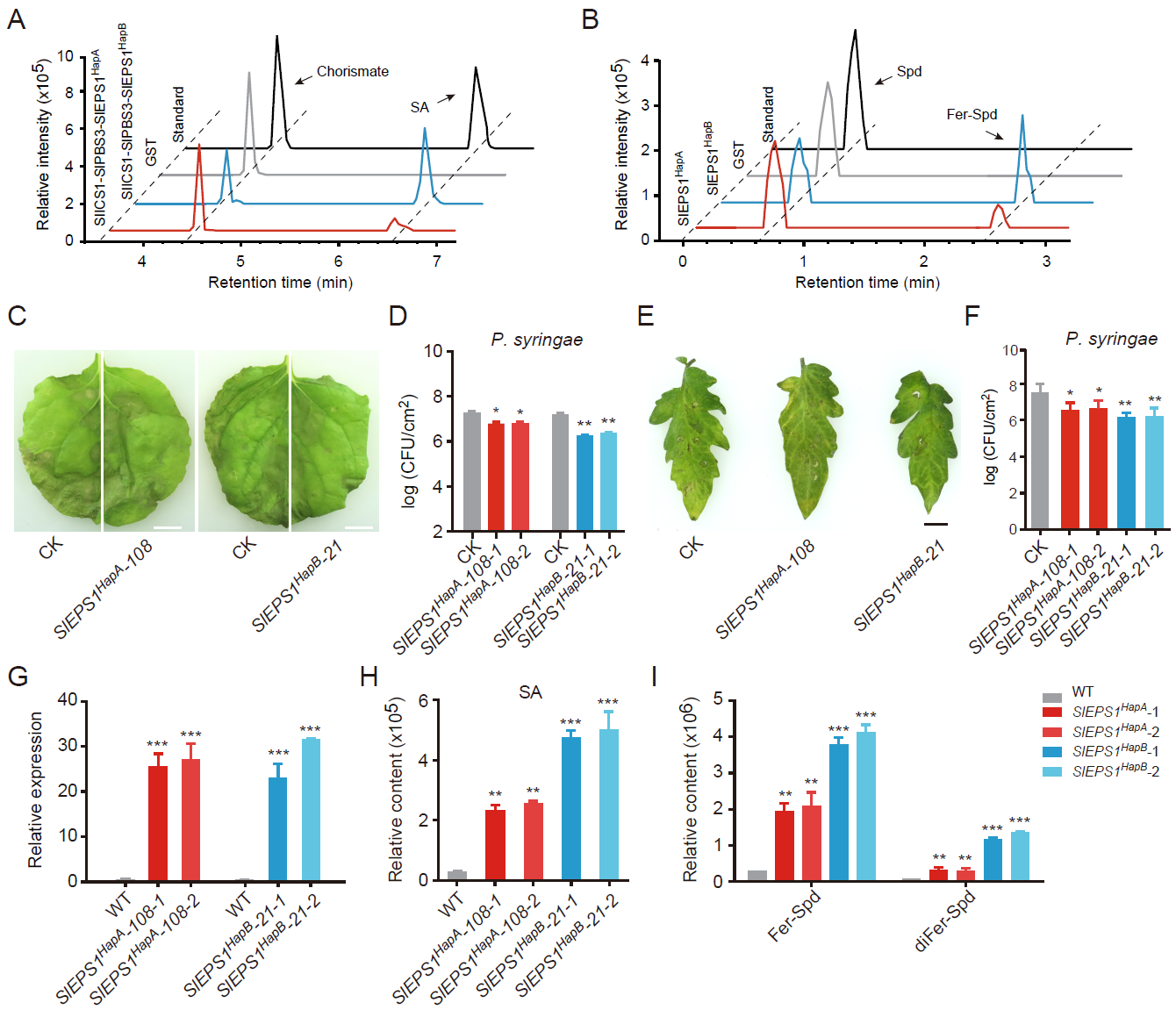


**Figure S7. Functional analysis of *SlEPS1*^HapA^*-108* and *SlEPS1*^HapB^*-21*.**

(**A**) High-performance liquid chromatography (HPLC) chromatograms of SA synthesis catalyzed by recombinant SlICS1-SlPBS3-SlEPS1^HapA^-108 and SlICS1-SlPBS3-SlEPS1^HapB^-21. Purified GST protein was used as a control.

(**B**) HPLC for recombinant SlEPS1^HapA^-108 and SlEPS1^HapB^-21 with spermidine and Fer-CoA. Purified GST protein was used as the control.

(**C**) Evaluation of disease resistance in transiently expressed *SlEPS1*^HapA^*-108* and *SlEPS1*^HapB^*-21* *N. benthamiana* leaves. Photographs were taken at 4 d after inoculation with *Pst DC3000*. d, day. Scale bar = 1 cm.

(**D**) Comparisons of bacterial growth in transiently expressed *SlEPS1*^HapA^*-108* and *SlEPS1*^HapB^*-21* *N. benthamiana* leaves at 4 d after inoculation.

(**E**) Assessment of disease resistance in transiently expressed *SlEPS1*^HapA^*-108* and *SlEPS1*^HapB^*-21* tomato leaves. Photographs were taken at 4 d after inoculation with *Pst DC3000*. Scale bar = 1 cm.

(**F**) Analysis of bacterial growth in transiently expressed *SlEPS1*^HapA^*-108* and *SlEPS1*^HapB^*-21* tomato leaves at 4 d after inoculation.

(**G**) Expression analysis in *SlEPS1*^HapA^*-108-OE* and *SlEPS1*^HapB^*-21-OE* lines with WT tomato plants as the control. OE, overexpression lines; WT, wild-type.

(**H and I**) Bar plots of the SA (**H**), and Fer-Spd and diFer-Spd (**I**) content in *SlEPS1*^HapA^*-108-OE* and *SlEPS1*^HapB^*-21-OE* lines with WT tomato plants as the control.


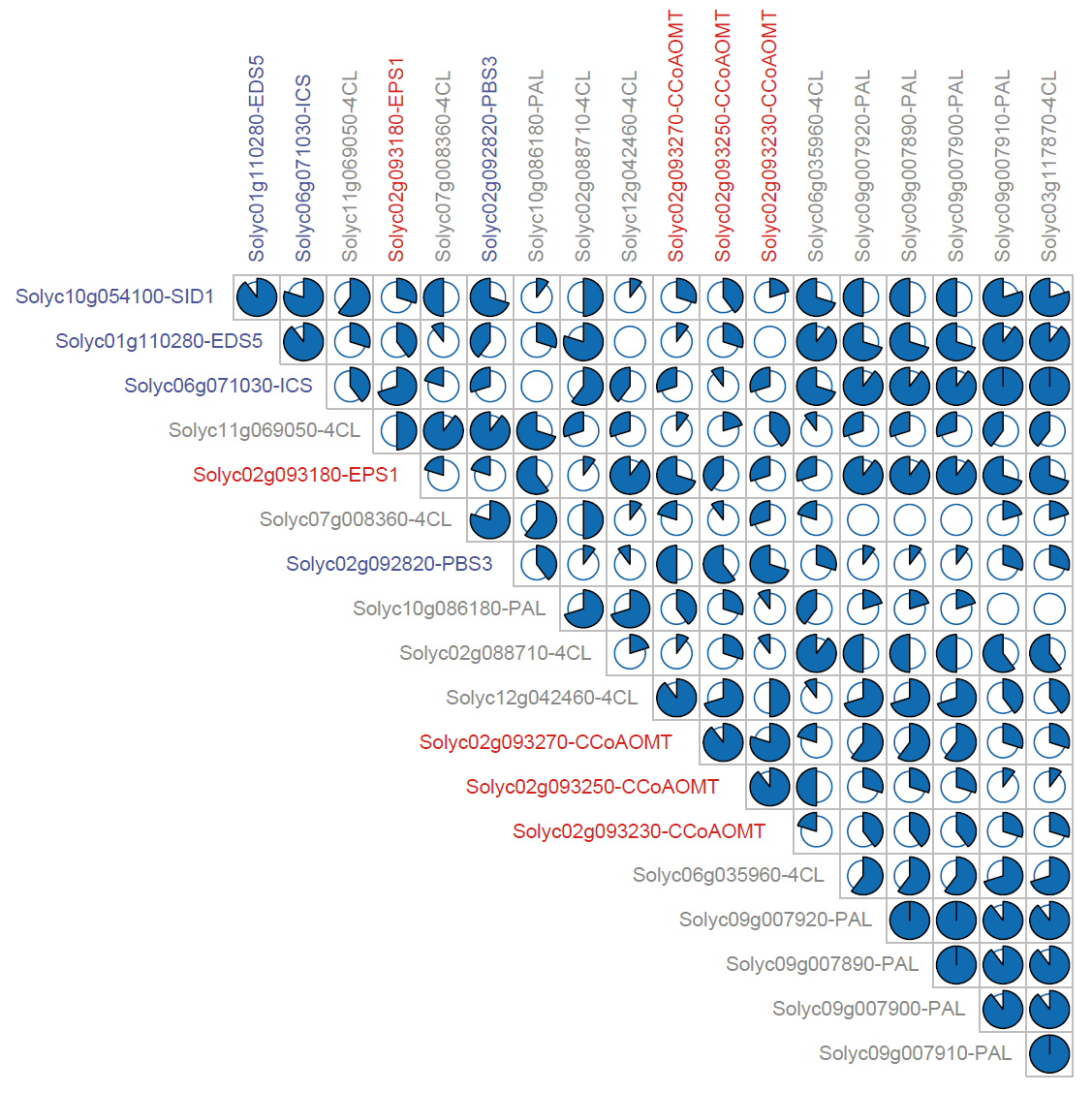


**Figure S8. Co-expression network map of BGC2 components and synthesis genes of SA and phenolamide.**


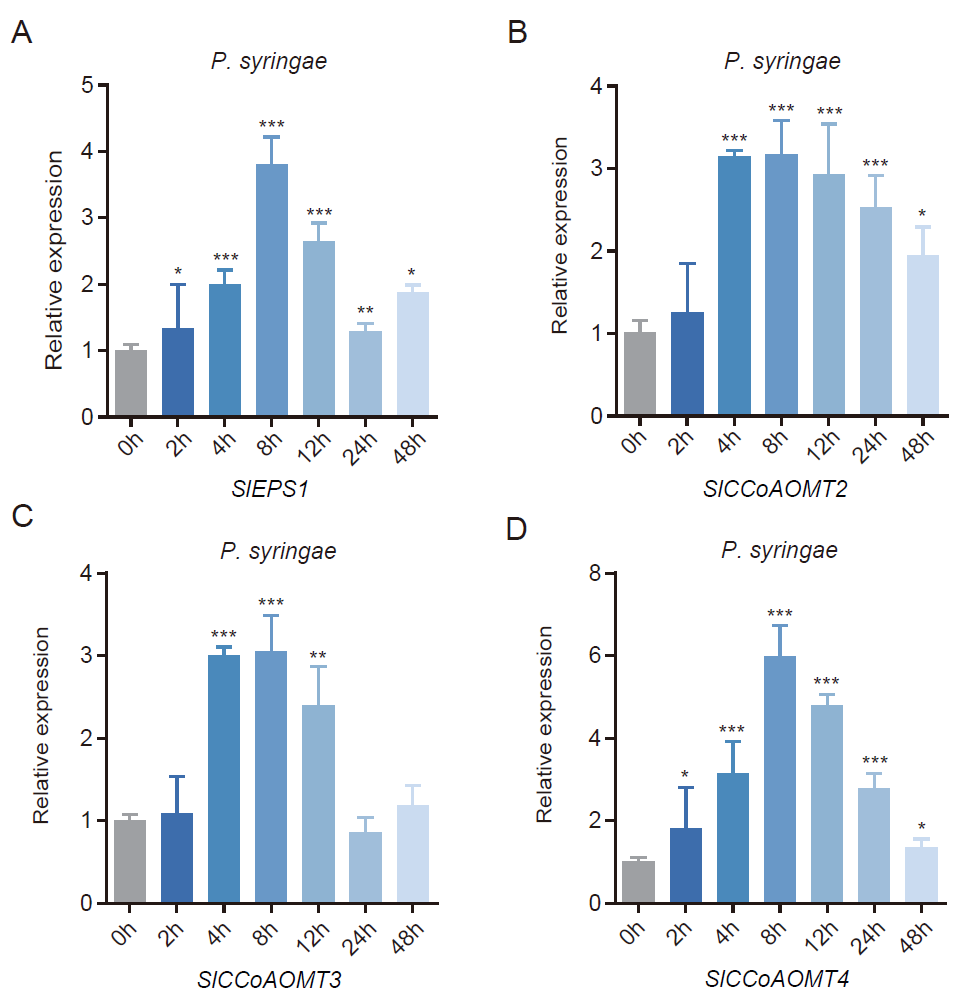


**Figure S9. Upregulation of the BGC2 after inoculation.**

Expression analysis for *SlEPS1* **(A)***, SlCCOAOMT2* **(B)***, SlCCOAOMT3* **(C)** and *SlCCOAOMT4* **(D)** in response to *Pst DC3000* infection.


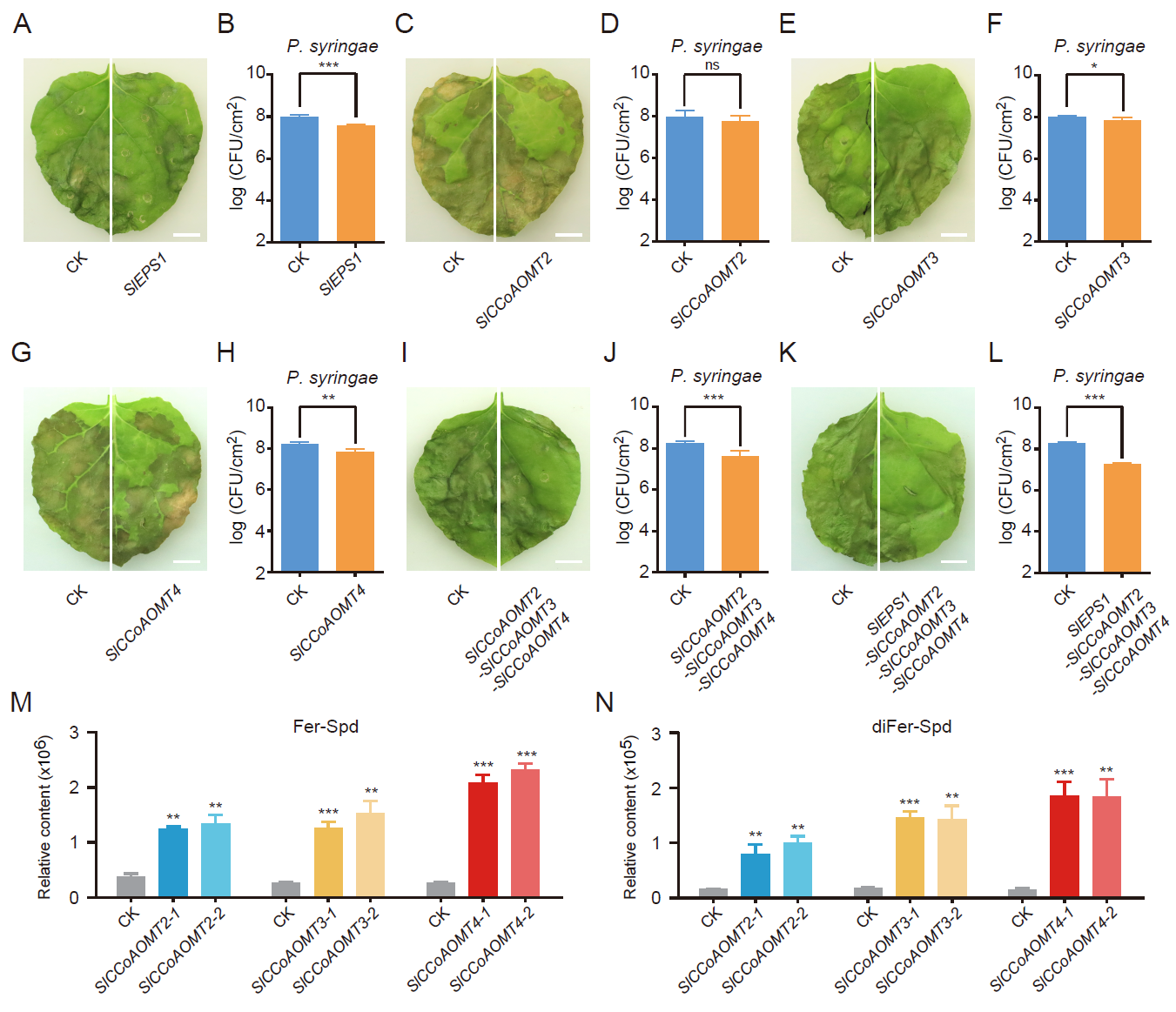


**Figure S10. Analysis of antimicrobial defense and bacterial colonization patterns in *N. benthamiana* leaves with transient co-expression of BGC2 gene cluster components following *Pst DC3000* infection.**

Evaluation of disease resistance in transiently expressed *SlEPS1* (**A**)*,* *SlCCoAOMT2* (**C**)*, SlCCoAOMT3* (**E**)*, SlCCoAOMT4* (**G**)*, SlCCoAOMT2-SlCCoAOMT3-SlCCoAOMT4* (**I**)*, SlEPS1-SlCCoAOMT2-SlCCoAOMT3-SlCCoAOMT4* (**K**) *N. benthamiana* leaves. Photographs were taken at 4 d after inoculation with *Pst DC3000*. d, day. Scale bar = 1 cm. Comparisons of bacterial growth in transiently expressed *SlEPS1* (**B**)*,* *SlCCoAOMT2* (**D**)*, SlCCoAOMT3* (**F**)*, SlCCoAOMT4* (**H**)*, SlCCoAOMT2-SlCCoAOMT3-SlCCoAOMT4* (**J**)*, SlEPS1-SlCCoAOMT2-SlCCoAOMT3-SlCCoAOMT4* (**L**) *N. benthamiana* leaves at 4 d after inoculation. (**M and N**) Accumulation of Fer-Spd (**M**) and diFer-Spd (**N**) in *N. benthamiana* leaves transiently overexpressing *SlCCoAOMT2, SlCCoAOMT3,* and *SlCCoAOMT4*, respectively, with control vector CK.


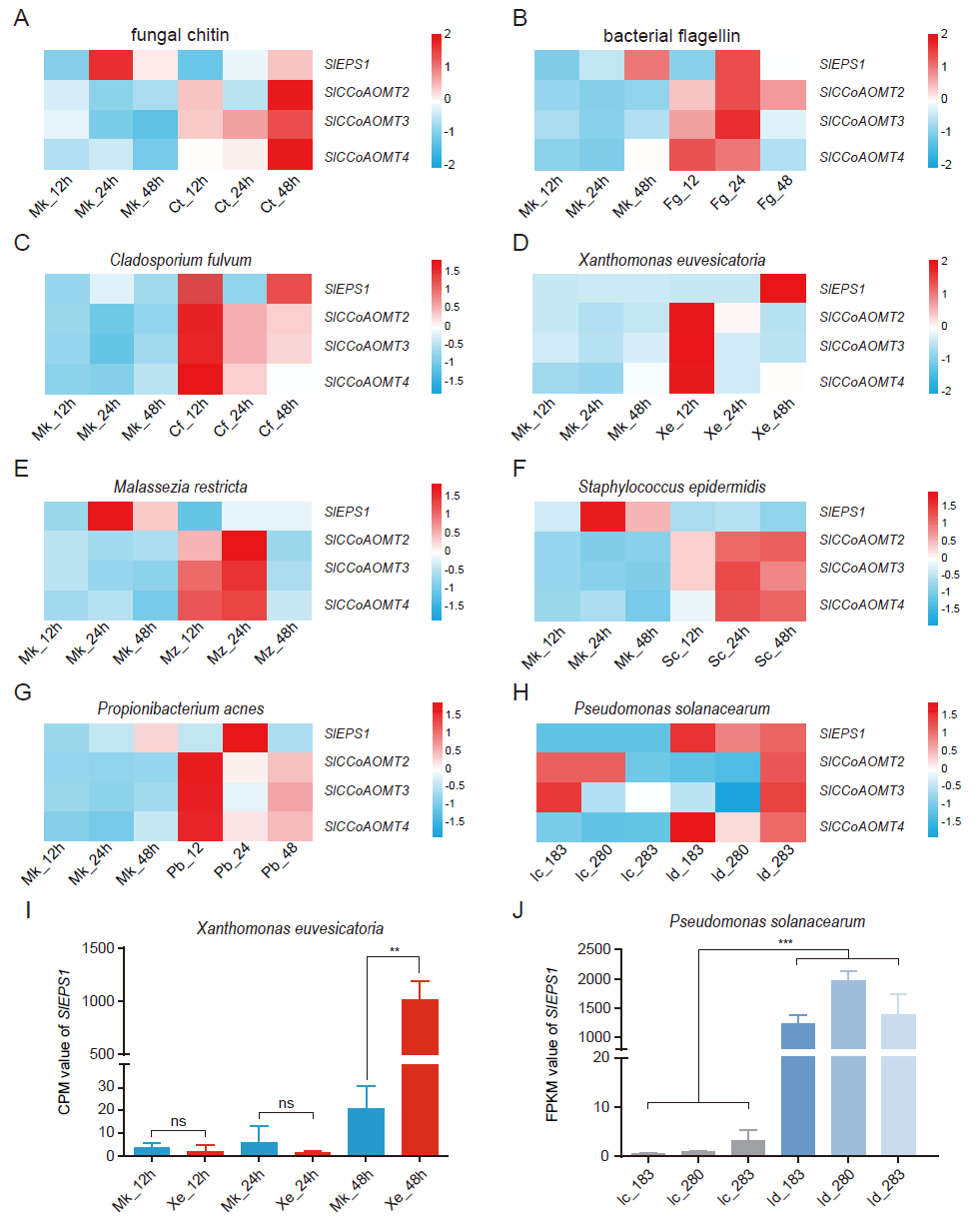


**Figure S11. Expression patterns of BGC2 component genes in tomato following induction by different pathogens.**

(**A-G**) Induced expression profiles of BGC2 component genes 12h, 24h, and 48h after inoculation with fungal chitin (**A**), bacterial flagellin (**B**), *Cladosporium fulvum* (**C**), *Xanthomonas euvesicatoria* (**D**), *Malassezia restricta* (**E**), *Staphylococcus epidermidis* (**F**), *Propionibacterium acnes* (**G**). Mock, MK; chitin, Ct; flagellin, Fg; *Cladosporium fulvum*, Cf. *Xanthomonas euvesicatoria*, Xe, *Malassezia restricta*, Mz, *Staphylococcus epidermidis*, Sc, *Propionibacterium acnes*, Pb.

(**H**) Expression profiles of BGC2 component genes in different tomato varieties before and after inoculation with *Pseudomonas solanacearum*. Leaf control, lc; Leaf disease, ld.

(**I and J**) Expression levels of *SlEPS1* gene 12h, 24h, and 48h after inoculation with *Xanthomonas euvesicatoria* (**I**), and *Pseudomonas solanacearum* (**J**).


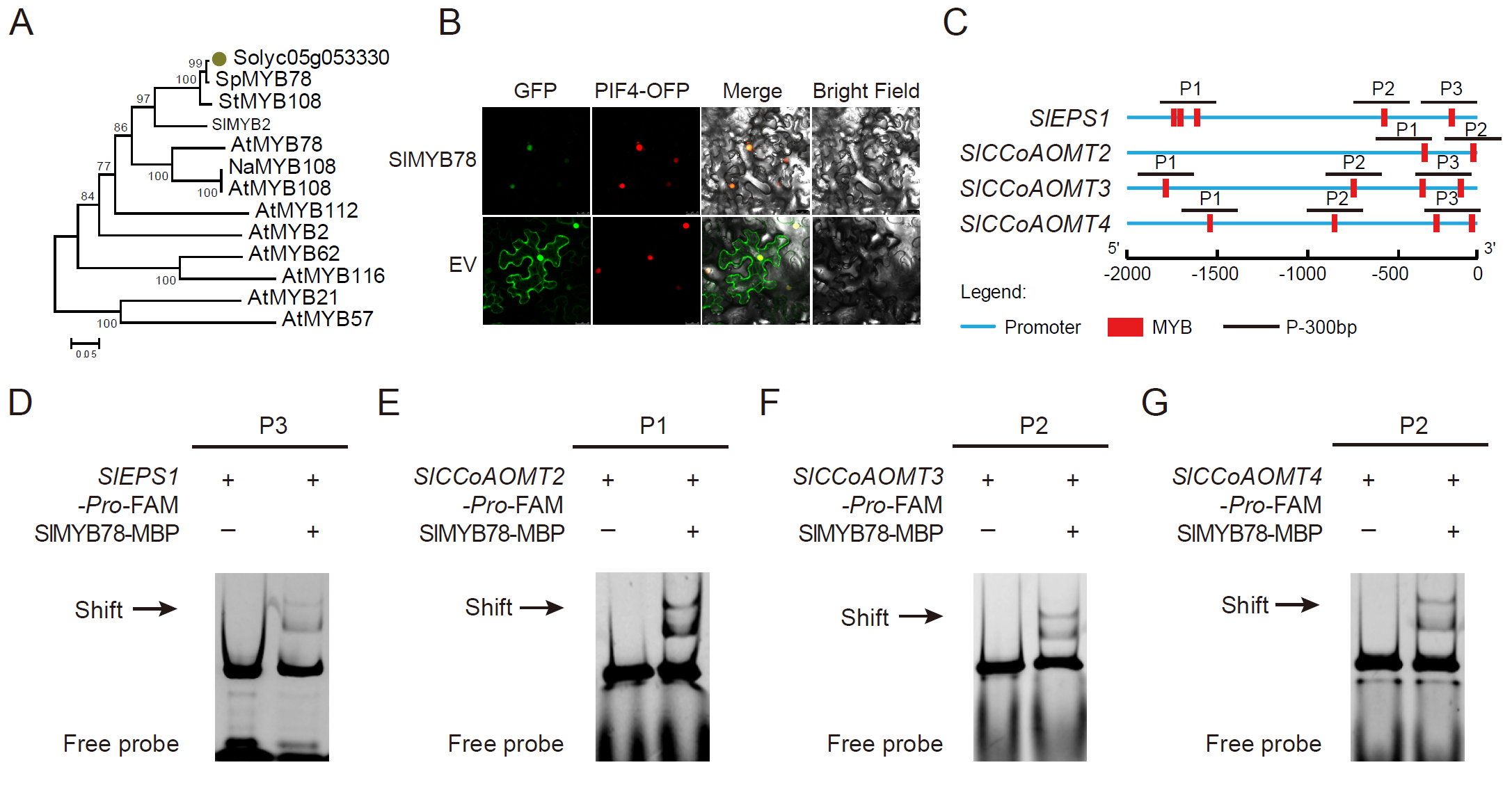


**Figure S12. Analysis of the *SlMYB78* and BGC2.**

**(A)** Neighbor-joining tree of SlMYB78 and related MYB transcription factor.

**(B)** Subcellular localization of SlMYB78 in *N. benthamiana*. Scale bar, 25μm. EV, empty vector.

**(C)** Promoter analysis of the phenolamide gene cluster BGC2. Schematic representation of the BGC11 and BGC7 promoter, with the position of the conserved MYB-box motifs indicated.

(**D-G**) EMSA analysis of the in vitro binding of MYB78 protein to the MYB-binding cis-elements on the BGC2 promoter. P1 to P3 are the DNA probes for EMSA. Binding of SlMYB78 to MYB-box motifs P1 to P3 as detected by EMSA. The labeled probes were incubated with SlMYB78-MBP. The bands corresponding to DNA-protein complexes (shift) and free probes are indicated by arrows. ‘‘–” and ‘‘+” indicate the absence and presence of the corresponding proteins or probes, respectively. Each experiment was repeated twice.


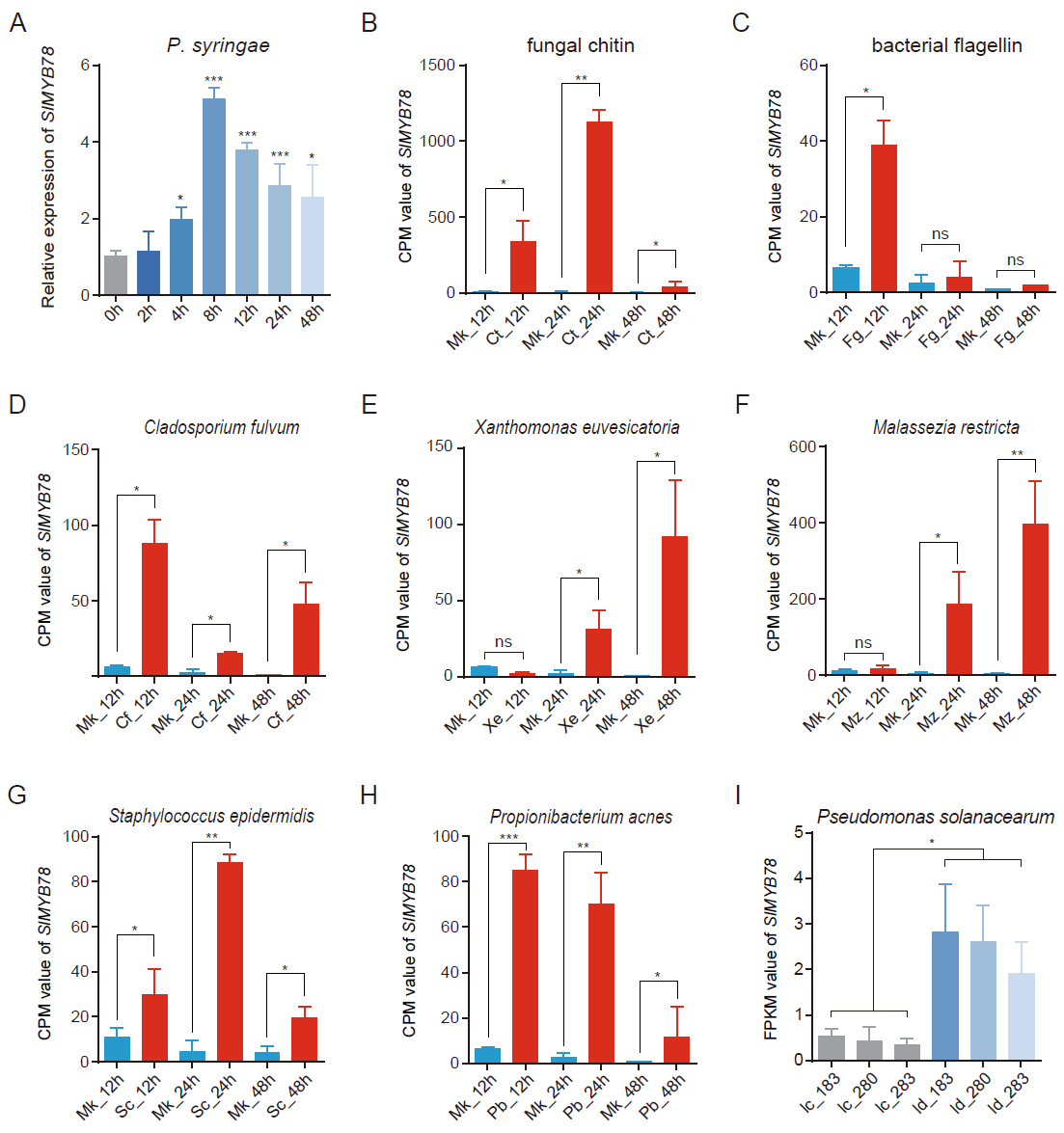


**Figure S13. Upregulation of the *SlMYB78* after inoculation.**

**(A)** Expression analysis for *SlMYB78* in response to *Pst DC3000* infection.

(**B-H**) Expression analysis of *SlMYB78* component genes 12h, 24h, and 48h after inoculation with fungal chitin (**B**), bacterial flagellin (**C**), *Cladosporium fulvum* (**D**), *Xanthomonas euvesicatoria* (**E**), *Malassezia restricta* (**F**), *Staphylococcus epidermidis* (**G**), *Propionibacterium acnes* (**H**).

(**I**) Expression levels of *SlMYB78* in different tomato varieties before and after inoculation with *Pseudomonas solanacearum*.
